# Pan-cancer analysis and the oncogenic role of Glypican 1 in hepatocellular carcinoma

**DOI:** 10.1038/s41598-024-66838-9

**Published:** 2024-07-09

**Authors:** Li Cao, Fang Li, Shuang Cai, Jinyuan Zhang, Chen Guo, Sadiq Ali, Jing Zhou, Xintao Jing, Xiaofei Wang, Yannan Qin, Fei Wu

**Affiliations:** 1 Comprehensive Breast Care Center, The Second Affiliated Hospital of Xi’an Jiaotong University, Xi’an, 710000 Shaanxi People’s Republic of China; 2 Department of Cell Biology and Genetics, School of Basic Medical Sciences, Xi’an Jiaotong University, Xi’an, 710061 Shaanxi People’s Republic of China; 3 Biomedical Experimental Center of Xi’an Jiaotong University, Xi’an, 710061 Shaanxi People’s Republic of China; 4 Institute of Genetics and Development Biology, Translational Medicine Institute, Xi’an Jiaotong University, Xi’an, 710301 Shaanxi People’s Republic of China; 5 Department of Oncology, The Second Affiliated Hospital of Xi’an Jiaotong University, Xi’an, 710000 Shaanxi People’s Republic of China

**Keywords:** GPC-1, Pan-cancer analysis, HCC, Proliferation, Apoptosis, Cancer, Cell biology

## Abstract

Recent studies indicate that Glypican 1 (GPC-1) is aberrantly expressed and plays a key role in certain cancers, but little is known in the hepatocellular carcinoma. Raw data from TCGA, GTEx and TIMER databases were utilized to comprehensively analyze GPC-1 expression landscape in pan-cancer, and the biological function of GPC-1 was investigated in liver cancer cells. The results revealed that GPC-1 is highly expressed in HCC, negatively correlated with survival, and also positively correlated with immune infiltration and clinical stage. Furthermore, GPC-1 promoted cell proliferation and inhibited apoptosis in the HCC cell lines. WGCNA analysis and HCCDB database revealed that Akt acted as a key molecule related to GPC-1, influencing biological functions and regulating cell malignant behaviors via the AKT signaling pathway. In conclusion, our findings provide a relatively comprehensive understanding of the oncogenic role of GPC-1 in HCC, implying that GPC-1 could serve as an innovative therapeutic target.

## Introduction

Cancer is a major public health problem worldwide and is considered to be the leading cause of death worldwide, contributing to rapidly increasing global morbidity and mortality^1^. According to global cancer statistics, there were 19,292,789 new cancer cases and 9,958,133 deaths due to various cancers in 2021^2^. Cancer treatments, including surgery, chemotherapy, radiotherapy, and targeted therapy, have greatly improved over the past few decades, but many types of tumors including liver cancer, remained incurable due to the lack of effective therapeutic targets. Hepatocellular carcinoma (HCC) is the fourth leading cause of cancer-related death worldwide, and liver resection is the main treatment for cancer treatment, but the recurrence rate within 5 years is more than 60%, and the prognosis is still poor and unsatisfactory^3^. Therefore, there is an urgent need to find new and reliable therapeutic targets for early detection, diagnosis, and treatment of cancer.

Glypicans (GPCs) are proteoglycans with heparan sulfate chains which can be attached to the outer surface of the plasma membrane through glycosylphosphatidylinositol anchors^4^. GPCs contain six human glypican family members (GPC-1 to GPC-6), which can be further divided into two broad subfamilies, GPC-1, GPC-2, GPC-4, GPC-6, as well as GPC-3 and GPC-5. In the two subfamilies, 25% of the amino acids are identical^5^. As an important member of the GPCs family, Glypican 1 (GPC-1) is encoded by the human GPC1 gene that is located at 2q37.3. GPC-1 consists of 558 amino acids containing a secretory signal peptide (residues 1–23), N-terminal core protein (residues 24–474), C-terminal heparan sulfate attachment region (residues 475–530), and ending with a sequence of hydrophobic residues attached to the GPI anchor^6^. The glycoside core protein of GPC-1 is composed of a stable α-helical fold (α1–α14) and three major loops (L1, L2, and L3), of which Asn-79 and Asn-116 two residues can be modified by N-linked glycans^7,8^. In the C-terminal heparan sulfate attachment region of GPC-1, Ser-486, Ser-488, and Ser-490 are substituted with a cluster of three heparan sulfate (HS) chains^6^. As a ubiquitous proteoglycan molecule, GPC-1 is considered to be a ligand carrier or complex receptor, which is widely present on cell membranes and in extracellular matrices^9^.

Recent studies showed that GPC-1 was aberrantly expressed and played an important role in certain cancers. For example, GPC-1 was significantly increased in pancreatic ductal adenocarcinoma, and the downregulation of GPC-1 suppressed pancreatic cancer cell growth and influenced the signaling of members of the TGF-β family of growth factors^10^. Moreover, GPC-1 expression was significantly associated with the perineural invasion of pancreatic cancer and had certain prognostic significance for pancreatic cancer patients^11,12^. Some other studies also showed that the expression of GPC-1 was increased in colorectal cancer, prostate cancer, and breast cancer^13–15^. However, little is known about the expression levels and clinical relevance of GPC-1 in pan-cancer, especially its roles in hepatocellular carcinoma (HCC).

In this study, the expression landscape, prognostic analysis, immune infiltration, and clinical staging analysis of GPC-1 in pan-cancer were conducted based on the cBioPortal database, TCGA database, GTEx database, GTEx database, CCLE database, HPA database and TIMER database. The function of GPC-1 in HCC cell lines (97H and HUH7) was determined by cell proliferation assay, colony formation assay, apoptosis assay, and wound healing assay. Then, the weighted gene co-expression network analysis (WGCNA) and HCCDB database were employed to predict the related core genes and their functions in HCC. The results showed that the overexpression of GPC-1 promoted the proliferation of HCC cell lines and inhibited its apoptosis, accompanied by the upregulation of Bcl-2 expression and the downregulation of Bax expression, while knockdown of GPC-1 inhibited proliferation, promoted apoptosis, and changed the expression of Bcl-2 and Bax in 97H and HUH7. And the change expression of GPC-1 had no influence on wound healing assay. The findings of this study suggest that GPC-1 influenced the prognosis of patients with cancers and might provide new research directions for cancer treatment.

## Materials and methods

### Gene expression analysis

In order to obtain the mRNA levels of GPC-1 in the different cancer cell lines, we first mapped the gene expression across the pan-cancer via the publicly available cBioPortal for Cancer Genomics (https://www.cbioportal.org/), which supports the visualization and analysis of multidimensional cancer genome data^16^. Based on the complete transcriptome data (primary tumor and normal tissue) and clinicopathological information data obtained from TCGA and UCSC Xena (https://xena.ucsc.edu), the expression levels of GPC-1 in the different cancerous and adjacent tissues were determined to analyze. Considering the small number of normal samples in the TCGA database, the combination of samples from GTEx and the TCGA database allowed for pan analysis to investigate the differential expression of GPC-1 in human cancers^17^. The protein expressions of GPC-1 in human normal tissues and tumor tissues were validated via the Human Protein Altas (https://www.proteinatlas.org/). Related single-cell analysis was applied by the Tumor Immune Single-cell Hub (TISCH) web tool (http://tisch.comp-genomics.org/documentation/).

### Survival prognosis analysis

The correlation between GPC-1 expression and the prognosis of patients in several types of cancers was analyzed using a R software package using the data obtained from TCGA and UCSC Xena. To obtain survival map data, COX univariate analysis and Kaplan–Meier Plotter were used to analyze the relationship between the expression and prognosis^18,19^. The Hazard ratios (HRs) and 95% confidence intervals were calculated using univariate survival analysis. When the mean value and 95% lower value appeared on the right side of the dividing line (HR = 1), the molecule could be regarded as risk, while it could be considered a conservative molecule if the mean value and 95% upper value were both less than 1. For Kaplan–Meier (K–M) survival analysis, samples were divided into two groups based on the higher (50%) and lower (50%) cutoffs: high-expression group and low-expression group. Kaplan–Meier plots were conducted to explore the associations between the expression levels of GPC-1 and overall survival (OS), disease-specific survival (DSS), disease-free survival (DFI), and progression-free interval (PFI).

### Immune infiltration analysis

In order to analyze the infiltration levels of immune cells and stromal cells in pan-cancer, the R package was used to visualize the relationship between GPC-1 expression and immune or stromal scores. Tumor Immune Estimation Resource (TIMER), based on high-throughput sequencing (RNA-Seq expression profiling), is a synthesized resource for systematic analysis of immune infiltration between tumor types and adjacent normal tissues (https://cistrome.shinyapps.io/timer/)^20^. The quantity of B cells, neutrophils, CD4^+^ T cells, CD8^+^ T cells, macrophages, and dendritic cells were evaluated using the TIMER method. Moreover, CIBERSORT method, EPIC method, MCPCOUNTER method and ABS method were used to analyze the relationship between the expression levels and different immune cells (cancer-related fibroblasts, mast cells, etc.). Spearman’s correlation was used to figure out and examined the abundance of cells, and log_2_^TMP+1^ was used to distribute gene expression levels.

### Clinical phenotypes analysis

In order to further analyze the correlation of GPC-1 expression with cancer pathological stage, data from tumor cases patients and healthy controls were gathered from the UCSC Xena website (https://xena.ucsc.edu). The tumor tissues were matched to different clinical stages to evaluate the relationship between differential GPC-1 expression and clinical progression^21^. T-test was used in two groups, and ANOVA was used in three groups or more.

### Expression analysis of GPC-1 In HCC

UALCAN (http://ualcan.path.uab.edu/index.html) is an online web tool that can be used to analyze tumor transcriptome data based on TCGA datasets^22^. By using the UALCAN web tool, the RNA level of GPC-1 in tumor and the compared normal samples of HCC was validated and further investigated the expression of GPC-1 in HCC based on individual cancer stage, patient’s race, patient’s gender, patient’s age, patient’s weight, TP53 mutation status, histologic subtypes, and tumor stages. Moreover, based on UALCAN, the correlation between GPC-1 expression and patient survival was explored, and combined survival plots were used to evaluate the effects of GPC-1 expression level and body weight, race, tumor grade or gender on survival of HCC patients. *P* values were calculated automatically. HCCDB database contains abundant HCC clinical sample information. Based on HCCDB data (https://www.hccdatasph.cn/app/ihga)^23^, the expression of GPC-1 in clinical samples was detected and adjacent meta co-expression network was constructed.

### Cell culture and transfection

Human liver cancer cell lines (97H and HUH7) were derived from the Cell Bank (Shanghai Genechem Co., Ltd., Shanghai, China). These Cells were cultured in DMEM medium (Gibco BRL, NY, USA) supplemented with 10% FBS (Gibco) in a humidified atmosphere containing 5% CO_2_ at 37 °C. Small interfering RNAs (siRNAs) and overexpression plasmids were designed and synthesized by Gene Pharma (SGC, Shanghai, China) to alter the expressions of GPC-1. Sequences of siRNA were shown in Supplementary file: Table S1. After culturing liver cancer cells for 24 h, GPC-1 siRNAs or plasmids were instantaneously transfected into liver cancer cells using Jet Primer (Polyplus-transfection, Ilkirch, France) according to the manufacturer's protocol.

### Quantitative real-time reverse transcription PCR (RT-qPCR)

Total RNA was extracted from the cells using TRIzol reagent (Invitrogen, Carlsbad, CA, USA) according to the manufacturer's instructions. The RNA was reverse-transcribed into cDNA using Hifair® II 1st Strand cDNA Synthesis Kit (Yeasen Biotechnology, Shanghai, Co., Ltd.). RT-qPCR was performed using Hieff® qPCR SYBR Green Master Mix (Yeasen Biotechnology, Shanghai, Co., Ltd.) according to the manufacturer’s protocol. Results were normalized to glyceraldehyde-3-phosphate dehydrogenase (GAPDH) gene expression. All experiments were performed in triplicate. Primer sequences were uploaded in the Supplementary file: Table S2.

### Western blotting

HCC cell lines proteins were extracted with RIPA buffer (Wolsen, Xi'an, China) and were quantified by the BCA Protein Assay Kit (Beyotime, China). Equal amounts of protein solution were run on 10% SDS-PAGE gels, and then transferred to PVDF membranes. Next, skim milk (5%) was used to block the membranes for 1 h. According to the given molecular weight of protein, the blots were cut prior to hybridization with antibodies during blotting and followed by these membranes were incubated overnight with relevant primary antibodies at 4 °C. Details of primary antibodies were mentioned in the supplementary data file: Table S3. After that, these membranes were incubated for 1 h at room temperature using the homologous secondary antibodies. Chemiluminescence assay protein blots were visualized by enhanced Chemiluminescence assay with Syngene GBox (Syngene, UK). Images of all replicate blots performed and fuller-length, original, unprocessed blots were added to Supplementary Figure S6.

### Cell proliferation assay

97H and HUH7 cells were seeded into 96-well plates (5000 cells/well in 200 μL medium) and cultured for 24 h. Afterward, cells were treated with siRNA or plasmids and then refreshed to the medium after 4 h. Cell proliferation was monitored using a Cytation^TM^5 Cell Imaging Multi-Mode Reader (Biotech, Winooski, VT, USA) at 6 h, 30 h, 54 h, and 78 h time periods. Suspension cells were counted separately using a Cytation^TM^5 Cell Imaging Multi-Mode Reader with the appropriate Gen5 Image software version 3.08. Each experiment had five replicates and was repeated three times.

### Colony formation assay

After the intervention of 97H and HUH7 cells for 24 h, 500 cells were seeded in each well of the 12-well plate. The cells were cultured in DMEM medium for 14 days and the medium was changed every 3 days. Cell colonies were fixed with 4% paraformaldehyde for 30 min, and stained with 0.2% crystal violet for 30 min. Three parallel wells were used for each group.

### Flow-cytometry assay

The cells were collected after the intervention with siRNAs or plasmids, followed by washing three times with PBS and resuspended as single-cell suspensions. Then, these cells were stained with Annexin-V-FITC Apoptosis Detection Kit (Sigma-Aldrich, USA) according to the instructions. The level of apoptosis was measured and analyzed by flow cytometry (Agilent, Palo Alto, USA).

### Wound healing assay

97H and HUH7 cells were seeded in 6-well plates at a density of 5 × 10^5^ cells per well to reach 80–90% confluency the following day. Transfections with siRNA or plasmid were performed to alter the expression of GPC-1. After 4–6 h, wounds were created with a 10 µL pipette tip, and PBS was used to remove cell debris. They were cultured in serum-free medium for 72 h, during which the images were observed and captured using a 10× magnifying photomicroscope (Nikon Corporation).

### Gene co-expression networks

Co-expression networks have proved useful to describe the pairwise relationships between gene transcripts. Weighted gene co-expression network analysis (WGCNA) is a common bioinformatic analysis method used to identify susceptibility modules and genes by clustering similar gene expression patterns. Spearman’s correlation analysis was used to analyze the correlation between GPC-1 and genes in the LIHC FPKM expression matrix, and the co-expression network was constructed with significantly different genes (*P* < 0.001). Firstly, samples were clustered in order to assess the presence of obvious outliers. Secondly, an appropriate soft-threshold power β was selected to get the similarity of adjacent co-expression. Next, we converted the adjacency into a topological overlap matrix (TOM) to measure the network connectivity of all differentially expressed genes. Finally, hierarchical clustering and the clustering tree were constructed to identify the co-expression gene modules of GPC-1. Pearson correlation analysis was used, and *P* < 0.05 was considered statistically significant. The genes in modules of interest were extracted for further functional enrichment analysis. The Gene Ontology (GO) and Kyoto Encyclopedia of Genes and Genomes (KEGG) pathway enrichment analysis were performed using the gene module gene of the R package. GO was utilized to pinpoint distinctive biological characteristics^24^. KEGG pathway enrichment analysis was performed to identify functional attributes^25^. The threshold was set as count > 2 and *P* < 0.05.

### Statistical analysis

All experiments were repeated at least three times, and all experimental data were analyzed by using Prism GraphPad 7.0 software with the presentation in the form of mean ± SD. The student’s t-test or one-way ANOVA was used to detect the differences among groups. *P* < 0.05 was considered statistically significant.

## Results

### Expression landscape of GPC-1 in *pan*-*cancer*

**Figure 1:**
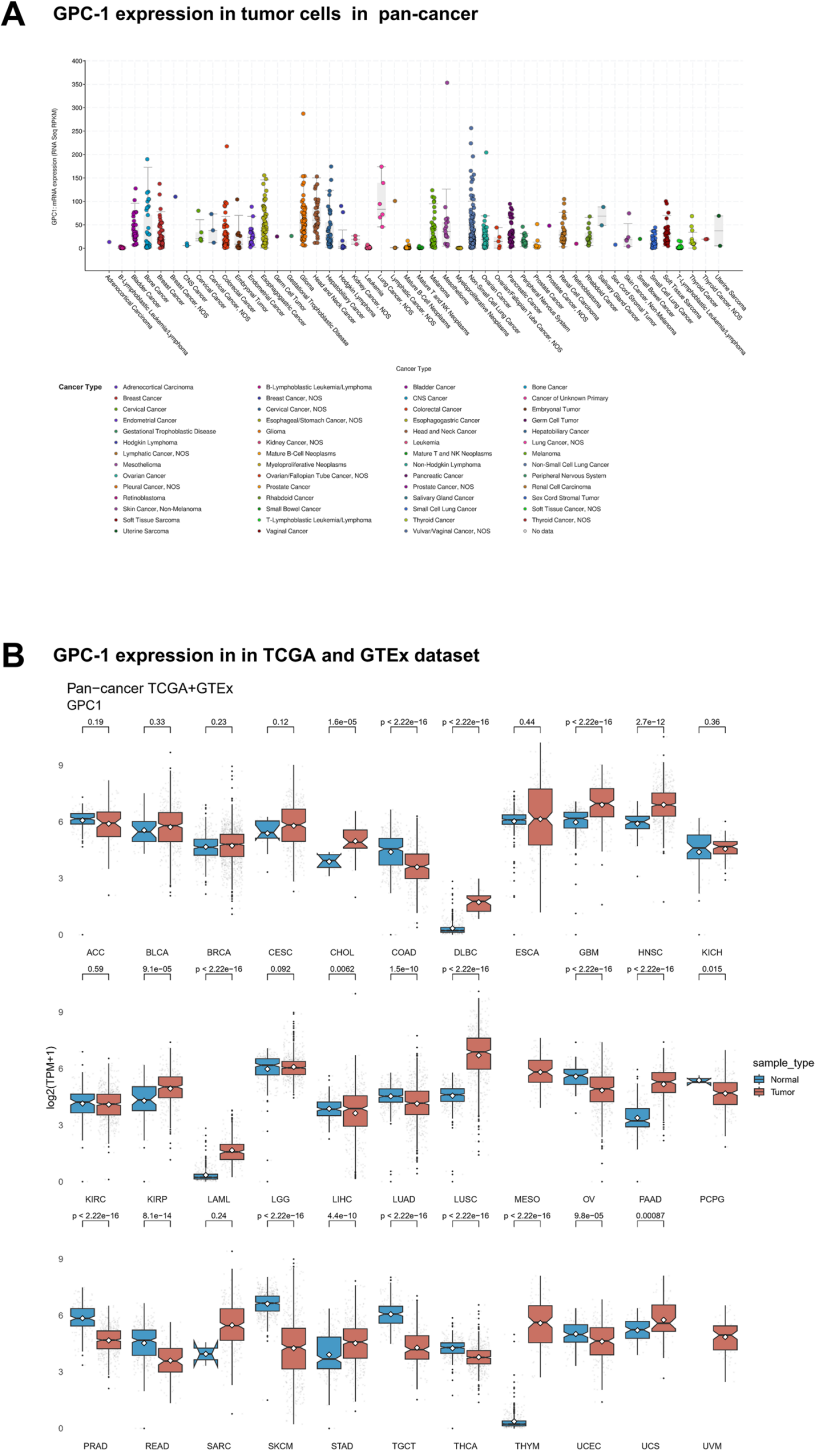
Expression landscape of GPC-1 in pan-cancer. (**A**) cBioPortal database analysis of the GPC-1 mRNA expression in tumor tissues. (**B**) GPC-1 expression in 33 human tumors was analyzed by integrating the corresponding normal tissues of the GTEx database and TCGA database (*P < 0.05, **P < 0.01, ***P < 0.001).

It has been widely proven that gene mutation is closely related to tumorigenesis, resulting in abnormal expression of the gene in cancers. The GPC-1 mRNA expression profiles in cancer patients were studied using the cBioPortal database. As shown in Fig. 1A, expression levels of GPC-1 were observed in most human tissues, including lung tissue, liver tissue, and kidney tissue. Furthermore, the expression pattern of GPC-1 in 33 tumors was analyzed by combining GTEx database and TCGA database. As shown in Fig. 1B, the expression level of GPC-1 in 12 or 10 tumor tissues was significantly higher or lower than that in their normal neighboring tissues (*P* < 0.05). GPC-1 expression was most significantly increased in lung squamous cell carcinoma (LUSC, *P* = 7.54e^−130^), Thymoma (THYM, *P* = 2.67e^−81^) and acute myeloid leukemia (LAML, *P* = 1.15e^−58^). Cancers with the most significant decreased expression of GPC-1 were found in Skin Cutaneous Melanoma (SKCM, *P* = 7.36e^−114^), followed by Testicular Germ Cell Tumors (TCGT, *P* = 1.46e^−51^) and Prostate adenocarcinoma (PRAD, *P* = 4.32e^−43^). Overall, the expression of GPC-1 in different cancer types have been rationalized differently in different tissues.

### Prognostic analysis of GPC-1 expression in *pan*-*cancer*

**Figure 2:**
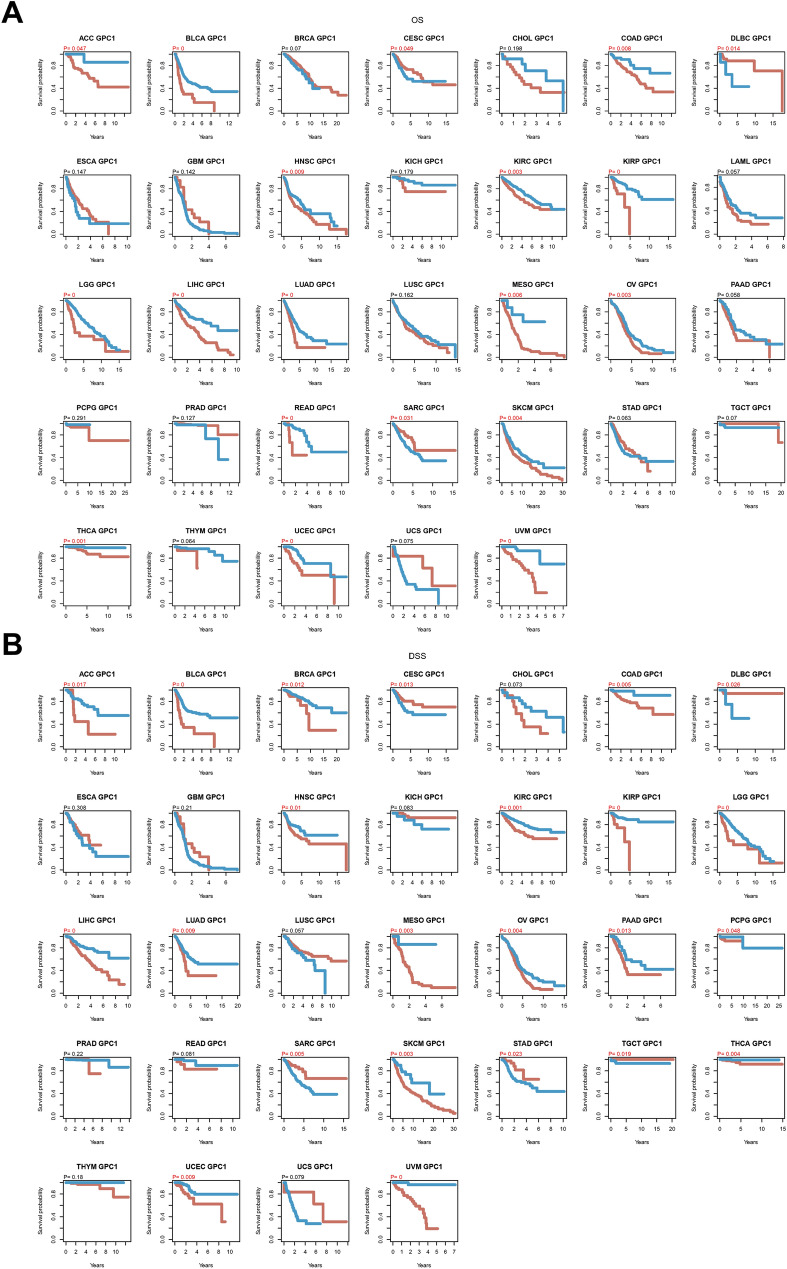
Pan-cancer analysis of the correlation between GPC-1 expression and survival prognosis. (**A**–**B**) Kaplan–Meier plotter analysis of the correlation between GPC-1 and prognosis. (**A**) OS, and (**B**) DSS.

**Figure 3:**
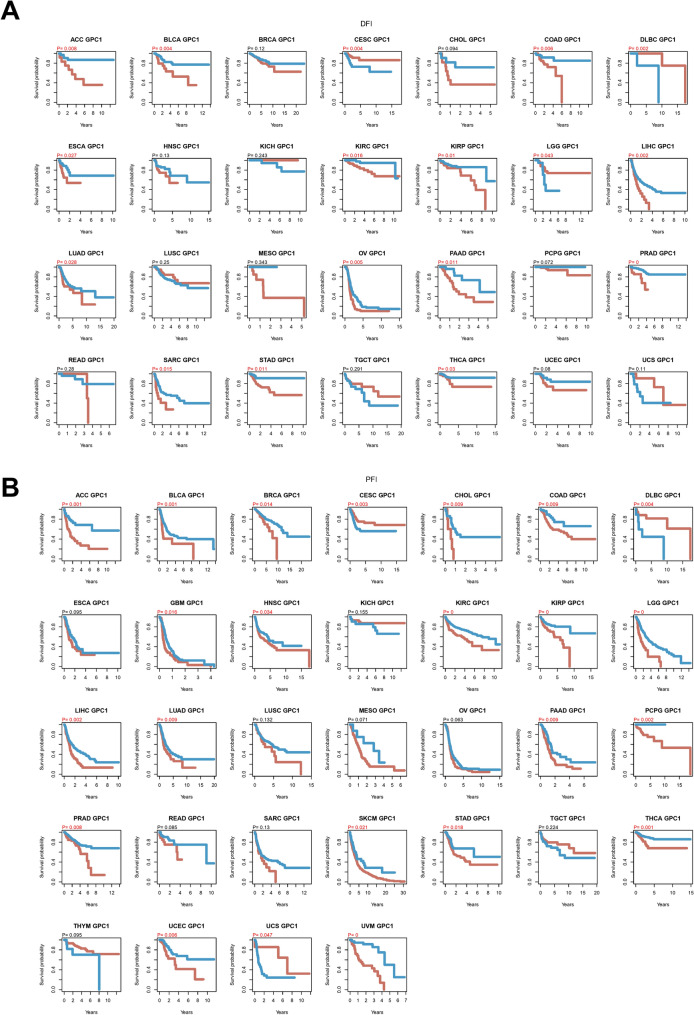
Pan-cancer analysis of the correlation between GPC-1 expression and survival prognosis**.** (**A**–**B**) Kaplan–Meier plotter analysis of the correlation between GPC-1 and prognosis. (**A**) DFI, and (**B**) PFI.

For investigation of the potential prognostic value of GPC-1 in different cancer types, COX univariate analysis and the Kaplan–Meier plotter were utilized. The results of OS analysis suggested that GPC-1 was a perilous factor for patients with LGG, UVM, LIHC, BLCA, LUAD, MESO, COAD, THCA, ACC, OV, and SKCM, while a protective factor for patients with LGG, UVM, HCC, BLCA (Figure S1). In addition, K-M survival plots were presented in 33 tumors, and the result showed that the expression of GPC-1 was positively correlated with the prognosis of 3 cancers and negatively correlated with the prognosis of 16 cancers with a *P* value less than 0.05 (Fig. 2A). In the 3 positively correlated cancers, GPC-1 was highly expressed in DLBC, while there was no significant difference in CESC and SARC. Among the 16 negatively correlated cancers, GPC-1 was highly expressed in HNSC, KIRP, and LIHC, and was low expressed in 7 cancers, including COAD, LUAD, and OV (Fig. 1B). Considering that there may be factors other than tumor death during the follow-up period, a comparison of DSS, DFI, and PFI was also respectively presented in Figs. 2B and 3, which also found generally consistent results.

### Immune infiltration analysis of GPC-1 in *pan*-*cancer*

**Figure 4:**
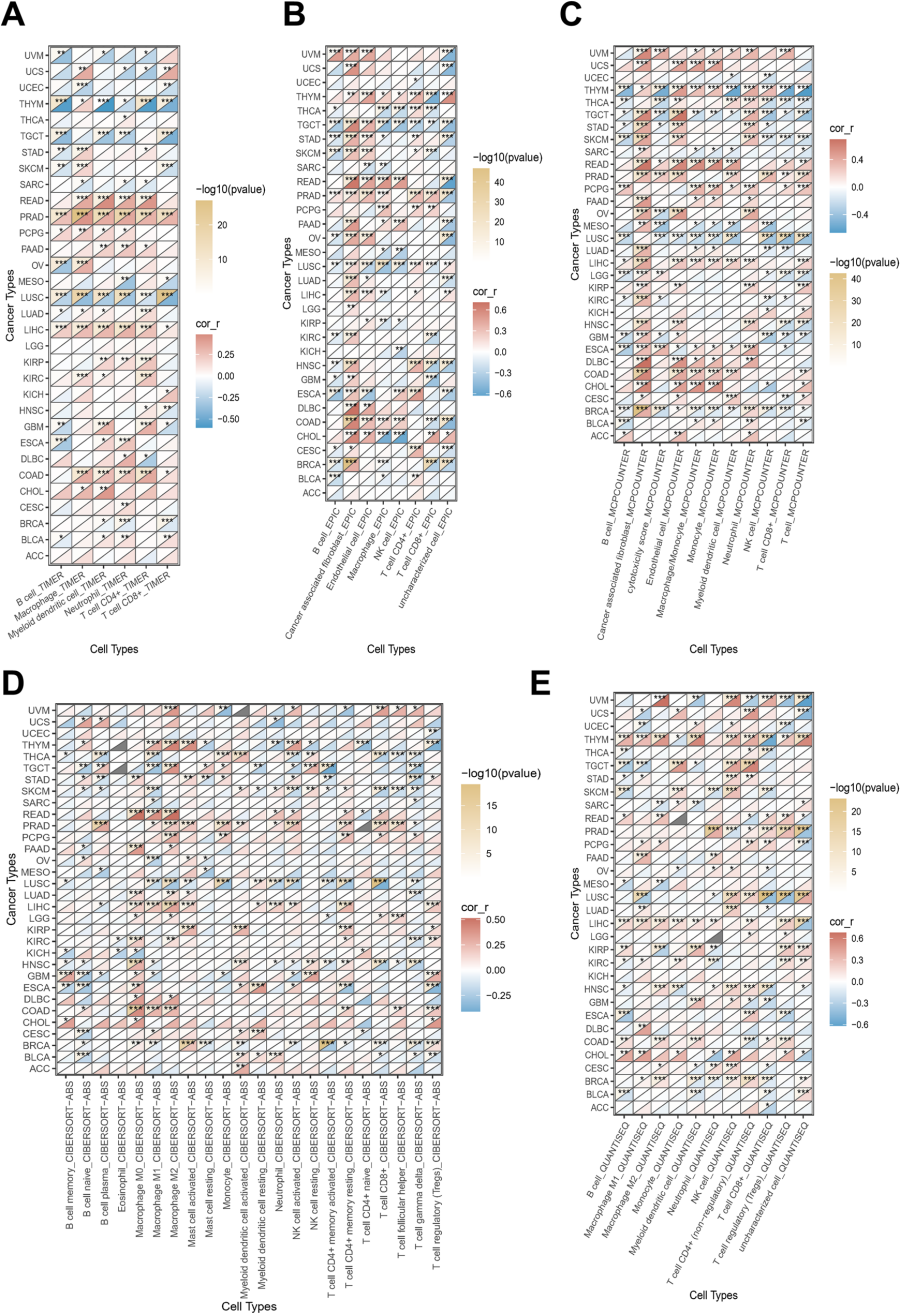
Correlation analysis of GPC-1 expression with immune infiltration. (**A**) The GPC-1 expression significantly correlated with the infiltration levels of various immune cells in the TIMER database. (**B**–**E**) The GPC-1 expression significantly correlated with the infiltration levels of various immune cells based on EPIC (**B**), MCPCOUNTER (**C**), ABS (**D**), and QUANTISEQ (**E**). ∗*P* < 0.05, ∗∗*P* < 0.01, and ∗∗∗*P* < 0.001.

Infiltrating immune cells play a crucial role in the predominant elements of the microenvironment and can influence tumorigenesis development or metastasis, thereby affecting the prognosis of cancer patients. We integrated TIMER, EPIC, MCPCOUNTER, ABS, and QUANTISEQ, five well-established algorithms designed to assess cross-tumor immune scoring. Based on the TIMER database, the results demonstrated that GPC-1 expression was significantly associated with the abundance of infiltrating immune cells: CD8^+^ T cells in 15 types of cancer, CD4^+^ T cells in 17 types of cancer, neutrophils in 19 types of cancer, myeloid dendritic cell in 18 types of cancer, macrophages in 16 types of cancer and B cells in 14 types of cancer (Fig. 4A). Notably, GPC-1 expression was positively correlated with immune scores of all six cell types in LIHC and PRAD, while negatively correlated with immune scores in all six cell types in LUSC (*P* < 0.05) (Fig. 4A). We further used the EPIC, MCPCOUNTER, ABS and QUANTISEQ tool to examine the relationship between GPC-1 expression and the infiltration of different types of immune cell subtypes. We discovered that the expression level of GPC-1 in pan-cancer was obviously correlated with most immunosuppressive cells, such as CAFs and TAFs (Fig. 4B–E). Moreover, single-cell data analysis was used to identify the main cell types that express the GPC-1 in cancer microenvironments, and the heatmap demonstrated the expression of GPC-1 of 54 cell types in 176 datasets using the TISCH. The results revealed that GPC-1 was mainly expressed in malignant cells, fibroblasts, and myofibroblasts (Figure S2). Our results indicate that GPC-1 might affect the development, prognosis, and therapy of cancers by associating with immune cells.

### Clinical phenotypes analysis of GPC-1 in *pan*-*cancer*

**Figure 5:**
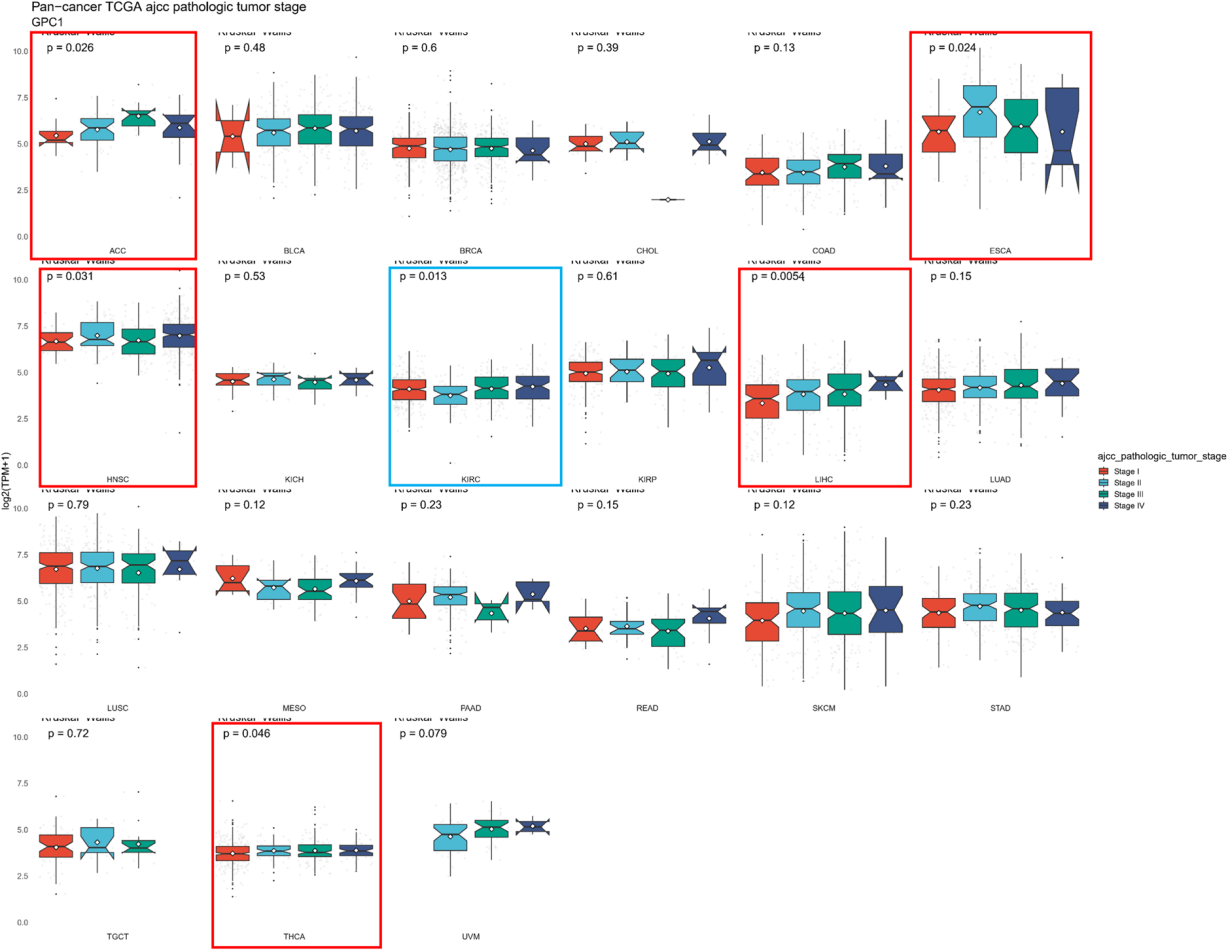
Correlation analysis of GPC-1 expression with Clinical Phenotypes.

To further investigate the association between GPC-1 expression and clinicopathological features in pan cancers, GPC-1 expression was observed in patients in 21 cancers with stages I, II, III, and IV according to the TCGA database. The results revealed that the expression of GPC-1 was correlated with the clinical stage in 6 kinds of tumors. In HCC, GPC-1 expression gradually increased with stage (*P* = 0.0054), and there was found a very strong positive correlation between stage I and stage II. In addition, GPC-1 expression was upregulated in ACC, ESCA, HNSC, and THCA from stage I to stage II with the progression of cancer stage, except in KIRC (*P* < 0.05) (Fig. 5). Overall, GPC-1 was highly expressed in HCC. All of these results implied that GPC-1 might play an important role in HCC.

### GPC-1 expression level in HCC

**Figure 6:**
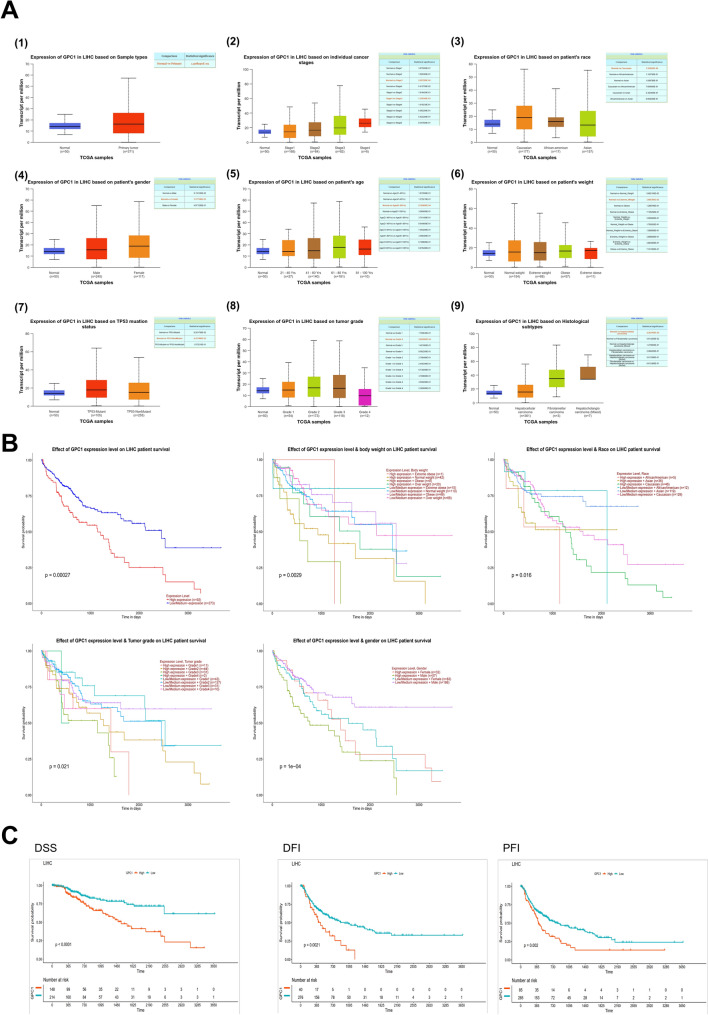
GPC-1 expression level and Survival analysis of GPC-1 in HCC. (**A**) GPC-1 expression level in HCC. (**B**–**C**) Survival analysis of GPC-1 in HCC.

To better characterize the relationship between GPC-1 and hepatocellular carcinoma, the mRNA level of GPC-1 normal and HCC tumor samples was validated using UALCAN web tool. The results showed that GPC-1 mRNA level was significantly (*P* < 0.05) elevated in the HCC samples compared with the normal group (normal n = 50, tumor n = 371), and GPC-1 showed significantly higher mRNA levels in stage 3, and there is a potential increase as the cancer progresses (Fig. 6A). Then, when GPC-1 expression was studied compared with patient race, gender, age, weight, TP53 mutation status, grade and histological subtype, there was no significant difference in the GPC-1 mRNA expression level. Based on datasets in HCCDB database, although there was no statistical significance, GPC-1 expression was slightly higher in HCC samples than in adjacent samples (Figure S3A).

### Survival analysis of GPC-1 in HCC

To better characterize the potential prognostic value of GPC-1 in HCC. The effect of GPC-1 expression level on HCC patient survival was validated by survival module of UALCAN web tool. The results showed that the overall survival was significantly shorter for HCC patients with higher GPC-1 expression level (n = 92) compared to those with low-medium GPC-1 expression level (n = 273) (*P* = 0.00027) (Fig. 6B). The expression of GPC-1 was significantly affected body weight (*P* = 0.0029), race (*P* = 0.016), tumor grade (*P* = 0.021), and gender (*P* = 1e−04) on HCC patient overall survivals (Fig. 6B). In addition, we also analyzed the relationship between the expression of GPC-1 and DSS, DFI and PFI by R software package survival (Fig. 6C), and the results showed high expression of GPC-1 was associated with a poorer prognosis. And an analysis based on the HCCDB15 dataset also consistent with the above analysis (Figure S3B). These results indicated that higher GPC-1 expression level associated with poorer HCC patients’ prognosis.

### Knockdown of GPC-1 inhibits proliferation and promotes apoptosis of HCC cell lines

**Figure 7:**
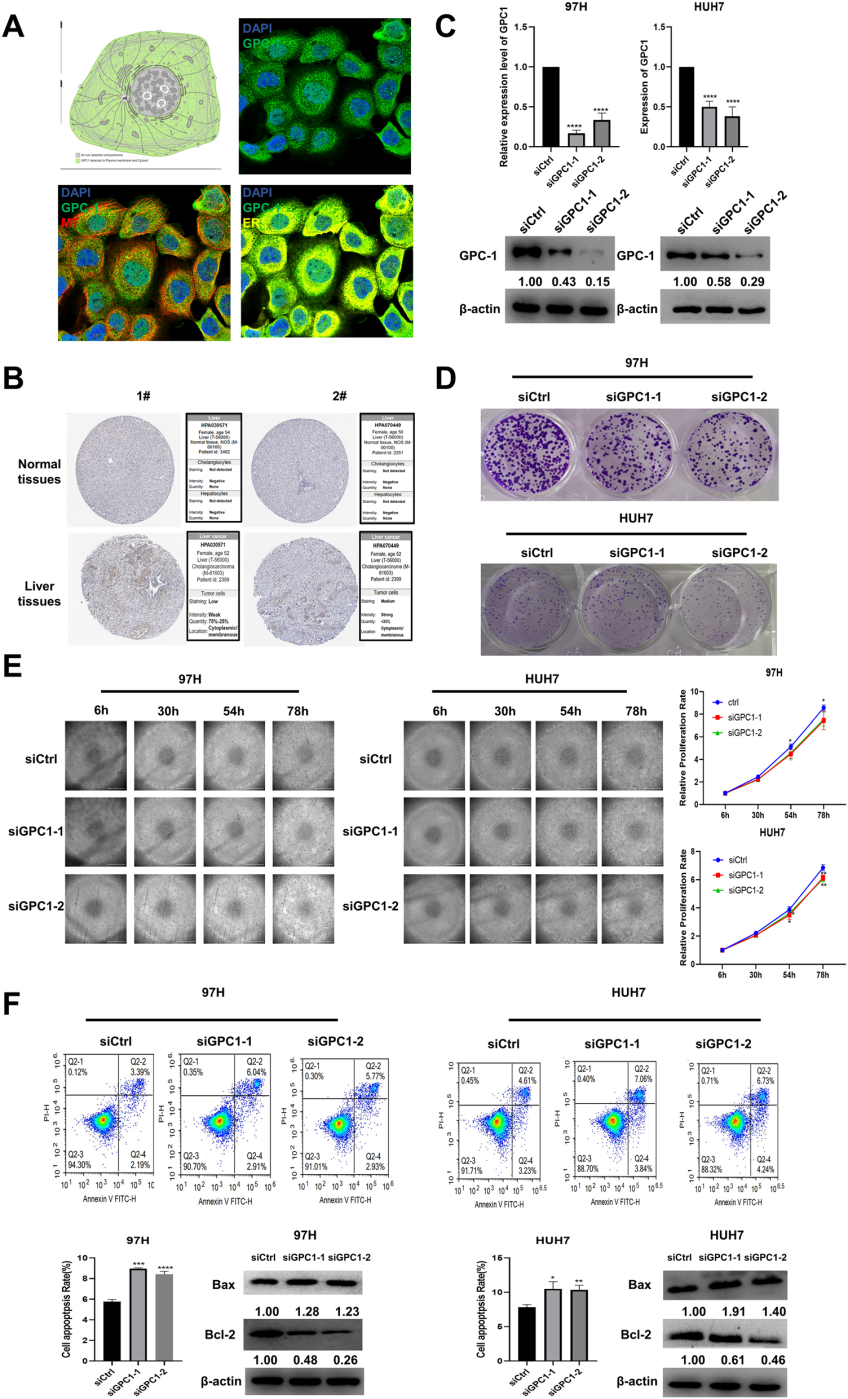
Knockdown of GPC-1 inhibited proliferation and promoted apoptosis of HCC cell lines. (**A**) GPC-1 was mainly expressed in Plasma membrane and Cytosol according to the Human Protein Atlas. (**B**) Protein expression of GPC-1 between LIHC tissues and normal tissues based on the HPA database. (**C**) RT-qPCR and western blotting were performed and revealed a significant reduce in the mRNA and protein levels of GPC-1 in liver cells. (**D**) Cell proliferation was detected by colony formation assay compared with the control group. (**E**) 97H and HUH7 cell numbers were examined by cell proliferation monitoring. (**F**) Flow-cytometry assay and western blotting were used to detect the cell apoptosis of GPC-1 downregulation. The uncropped images were presented in Supplementary Figure S6. (*P < 0.05, **P < 0.01, ***P < 0.001).

To further define the relationship of GPC-1 in HCC, the function of GPC-1 in 97H and HUH7 cells was examined. According to the Human Protein Atlas databases, GPC-1 was expressed in the plasma membrane and cytosol of the cell (Fig. 7A), and the protein level of GPC-1 was apparently higher in liver tumor tissues than in normal tissues (Fig. 7B). Two specific siRNAs (siRNA1, 2) targeting GPC-1 were designed and respectively transfected into 97H and HUH7 cell lines. The RNA and protein levels of GPC-1 were down-regulated in the cells (Fig. 7C). Moreover, colony formation assay and cell proliferation monitoring assay were used to examine cell viability and proliferation, and cell apoptosis was measured via flow cytometry assay. Our data demonstrated that the transient transfection of siRNA1 and siRNA2 suppressed the growth and promoted apoptosis of the cells, compared to control groups (Fig. 7D–F).

### Overexpression of GPC-1 promotes proliferation and inhibits apoptosis of HCC cell lines

**Figure 8:**
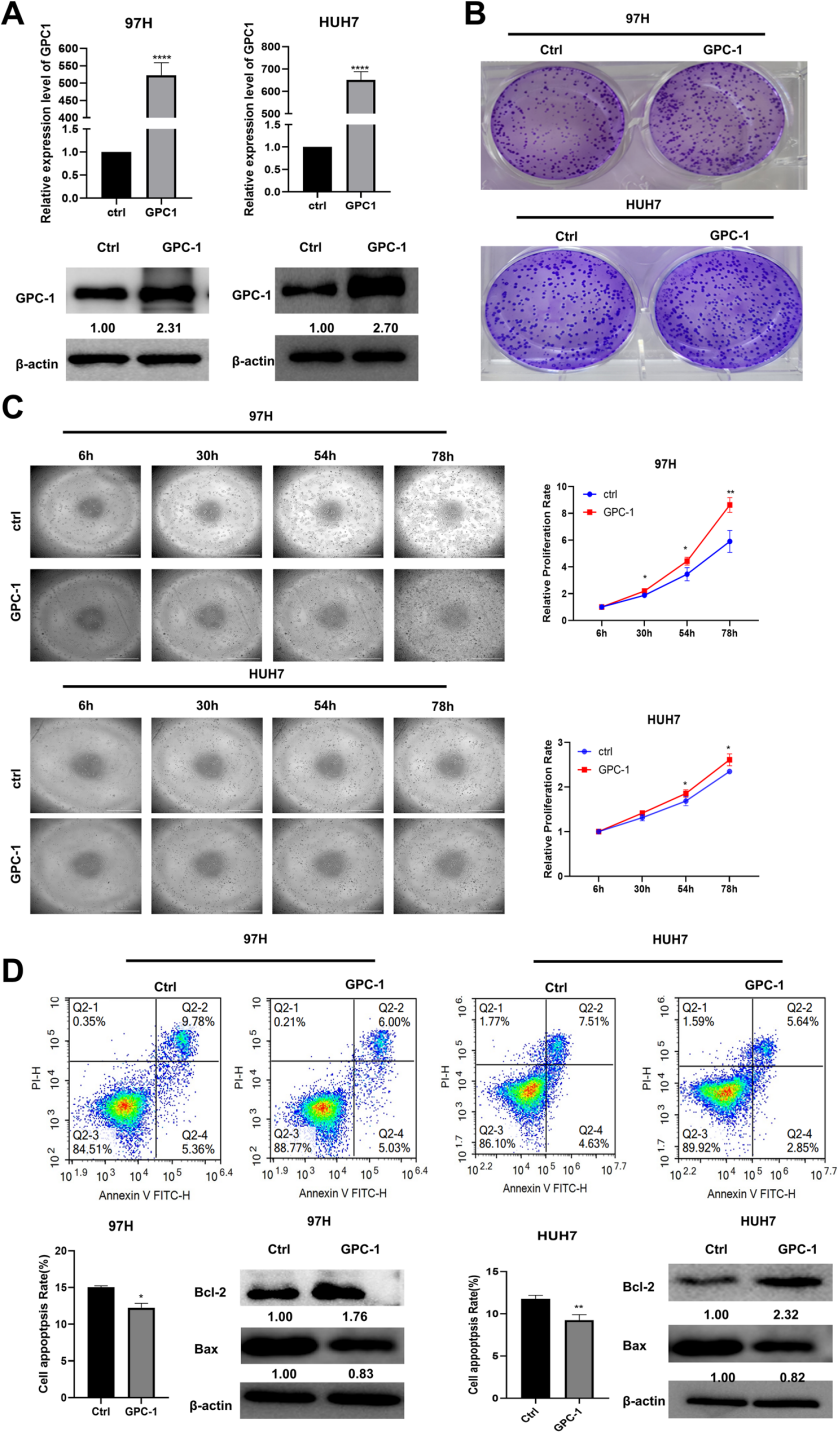
Overexpression of GPC-1 promoted proliferation and inhibited apoptosis of HCC cell lines. (**A**) RT-qPCR and western blotting were performed and revealed a significant increase in the mRNA and protein levels of GPC-1 in liver cells. (**B**) Colony formation assay was used to detect cell proliferation compared with the control group. (**C**) 97H and HUH7 cell numbers were examined by cell proliferation monitoring. (**D**) Flow-cytometry assay and western blotting were used to detect the cell apoptosis of GPC1-overexpressing. The uncropped images were presented in Supplementary Figure S6. (*P < 0.05, **P < 0.01, ***P < 0.001).

In contrast, the overexpression plasmid was then transfected into 97H and HUH7 cell lines to analyze cell growth. The results showed a higher proliferation rate in the cell proliferation monitoring assay and more colonies in the colony formation assay compared to that in control groups, suggesting that the overexpression of GPC-1 promoted liver cell viability and proliferation (Fig. 8A–C). Furthermore, flow-cytometry assay and western blotting were used to identify the cell apoptosis. The results showed that apoptosis was reduced in both 97H and HUH7 cells overexpressed with GPC-1 compared with the control group (Fig. 8D). Meanwhile, the wound healing assay indicated that overexpression of GPC-1 in 97H and HUH7 had no significant impact on cell migration (Figure S4).

### Prediction of the downstream genes of GPC-1 and their functions

**Figure 9:**
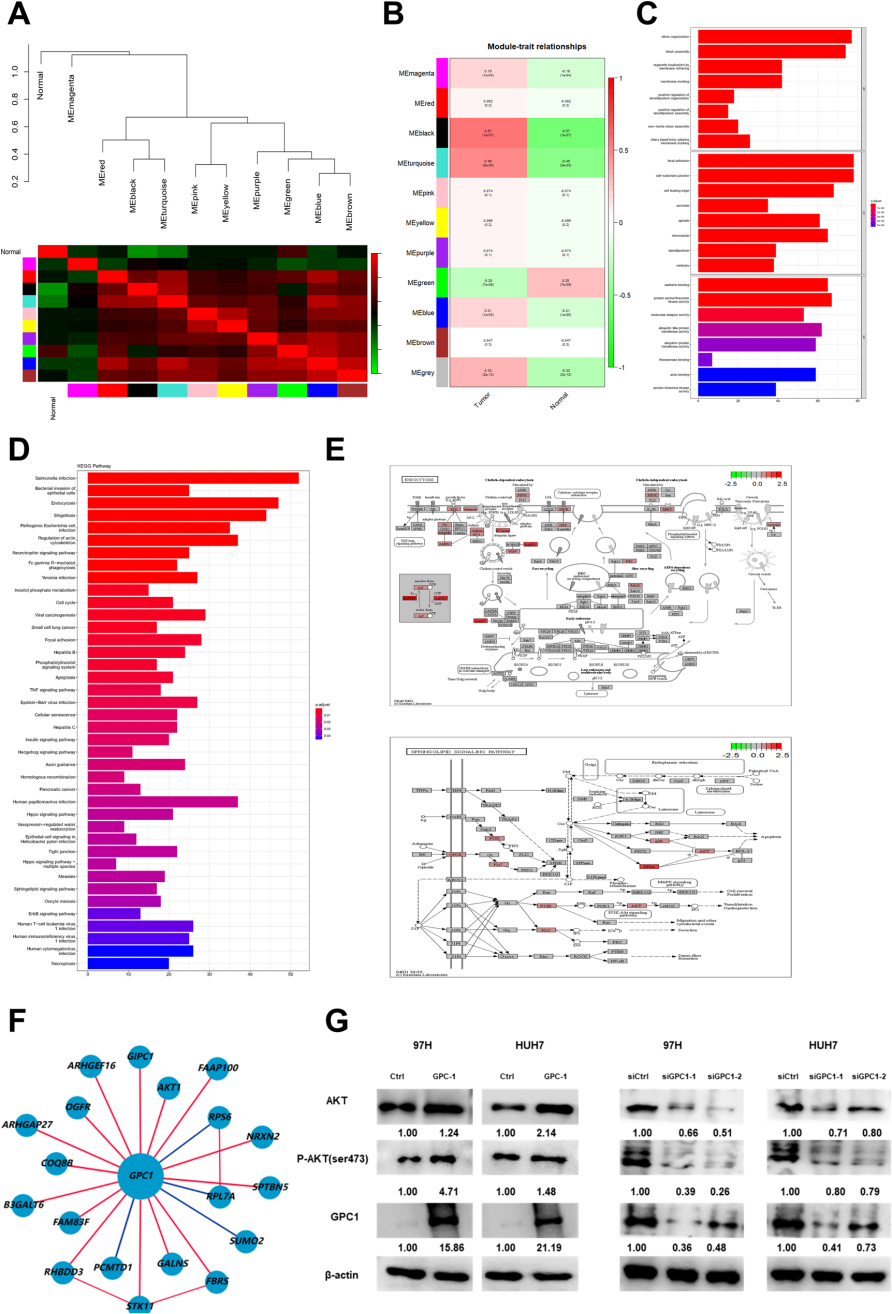
Prediction of the downstream genes of GPC-1 and their functions. (**A**) Module–clinical trait relationships of module eigengenes in LIHC. Module names are displayed on the left. The rows are colored based on the correlation of the module to the LIHC group: red for positive correlation and green for negative correlation. (**B**) Eigengene dendrogram and eigengene adjacency plot. In the adjacency plot, the green color represents low adjacency (negative correlation), while a red represents high adjacency (positive correlation). (**C**) Gene Ontology analysis of the genes involved in the turquoise module regarding biological process, cellular component, and molecular function. (**D**) KEGG enrichment analysis results of turquoise module genes. Pathway names are shown on the left. The X-axis represents the number of gene counts in the KEGG pathway. Color of the columns represented as the *P* value, which is displayed on the right side of the heatmap. (**E**) Pathway of endocytosis and sphingolipid signaling pathway. (**F**) Adjacent meta co-expression network of GPC-1 in HCCDB database. (**G**) The western blotting of AKT and phosphorylated‐AKT (ser473) upon knockdown and overexpression of GPC-1 in 97H and HUH7. The blots have been cropped to improve the conciseness and clarity of the display. The uncropped images were presented in Supplementary Figure S6.

All genes related to GPC-1 in HCC were calculated by Spearman’s correlation analysis, and the genes screening (*P* < 0.001) were analyzed by WGCNA (Table S4). In the WGCNA network, when the soft threshold power was determined to be 10 by the picksoft Threshold function, the fit index of the scale-free topology model reached 0.9, and the mean connectivity was close to 0, showing a relatively high average connectivity (Figure S5A). Next, eleven modules were identified based on average hierarchical clustering and dynamic tree clipping through the WGCNA R package (Figure S5B). The results of clustering the eigengenes showed that 10 modules could be clustered into 2 clusters, and 3 combinations (module blank and turquoise, module pink and yellow, and module blue and brown) showed high interactive connectivity (Fig. 9A). Then, 5 modules (MEblack, MEturquoise, MEgrey, MEblue, MEmagnta) were positively correlated with HCC, and one module (MEgreen) was negatively correlated with disease (*P* < 0.05) (Fig. 9B). Although the black module was the most correlated with pathological grade (*P* = 1e^−37^), the number of genes clustered was very small, so we chose the second correlated turquoise module (*P* = 2e^−23^) as a clinically important module for further analysis. GO analysis of genes in the turquoise module revealed that these genes were enriched in biological processes such as organelle localization through membrane tethering, focal adhesion, cadherin binding, and molecular adaptor activity (Fig. 9C). Among the KEGG terms, the result showed that GPC-1 was enriched with salmonella infection, endocytosis, and shigellosis with *P* value < 0.05, and these genes were mapped to neurotrophins signaling pathway, TNF signaling pathway, and sphingolipid signaling pathway (Fig. 9D). In addition, the visual analysis of endocytosis showed that GPC-1 was closely related to ARP23, RTK, GPCR and other molecules (Fig. 9E), and the visual analysis of sphingolipid signaling pathway showed that the expression of GPC-1 could regulate GPCR, Akt, p38 and other signaling molecules to affect cell apoptosis (Fig. 9E). Moreover, utilizing the HCCDB database, we constructed the adjacent meta co-expression network of GPC-1, identifying Akt as a key molecule associated with GPC-1 (Fig. 9F). Based on it, we hypothesize and verified that AKT signal pathway might modulate the GPC-1‐induced cell apoptosis and proliferation of 97H and HUH7 cells. The results showed that the expression of AKT and phosphorylated‐AKT(ser473) were significantly up-regulated after GPC-1 overexpression, whereas their levels decreased upon GPC-1 knockdown in 97H and HUH7 cells. These results suggested that GPC-1 may regulate malignant cell behaviors through the AKT signaling pathway (Fig. 9G). Taken together, these data supported that GPC-1 might influence biological function by regulating the cell proliferation and promoting malignant behavior through the AKT signaling pathway.

## Discussion

GPC-1 is a proteoglycan anchored to the surface of cell membranes, and there have been several publications reporting that GPC-1 participates in cell biological events, such as cell proliferation, differentiation, apoptosis, and organ development^26–28^. Currently, a growing number of studies have shown that GPC-1 is closely associated with clinical diseases (especially tumors)^15,29–31^. Those findings are in line with our analysis. It has not been confirmed that GPC-1 participates in the etiology of certain malignancies through a particular pathway, and more research is required to determine the precise molecular mechanism. Our study, based on the analysis of numerous databases, found that GPC-1 expression is upregulated in at least 12 common cancers and is associated with survival outcomes, as well as immune infiltration and clinical staging of several different types of cancer.

In order to accurately evaluate the expression pattern of GPC-1 in different tumor tissues or cancer cell lines, we first analyzed the cBioPortal data to accurately assess GPC-1 mRNA levels GPC-1 among 21 types of tumor tissues and then explored the expression of GPC-1 in 33 cancers with the combination of normal tissue data from GTEx database and TCGA database. Based on the analysis of multiple databases, our study found that GPC-1 was upregulated in CHOL, DLBC, and other 10 common cancers and downregulated in 10 cancers, suggesting that GPC-1 may have different functions in different types of cancer. In addition, survival prognosis, immune infiltration, and clinical phenotype analysis of GPC-1 expression in pan-cancer showed an association with its upregulated expression. Although our findings suggested that GPC-1 expression was associated with survival prognosis, clinical stage, and immune infiltration, we were unable to determine whether GPC-1 had an impact on patient survival as a result of immune infiltration. Further research on immune cell infiltration and GPC-1 expression in other cancer populations might provide additional insights into this question.

Recently, increasing evidence has shown that GPC-1 is a cancer-associated protein that would aid in the detection and monitoring of cancer development. A study has shown pancreatic cancer patients are absolutely specific and sensitive to distinguish healthy subjects and patients with a benign pancreatic disease from patients with early and advanced pancreatic cancer^32^. Truong's study has revealed that the highly glycosylated proteoglycan GPC-1 can represent a clinically relevant biomarker for the diagnosis of prostate cancer^33^. Jian Li and his colleague also found that plasma GPC-1 positive exosomes could be considered as a biomarker for colorectal cancer^34^. Furthermore, Wang and Chen’s studies research also suggested that high GPC-1 expression was closely associated with a poor prognosis in patients with HCC using bioinformatics analysis^35,36^. Consistent with these previous studies, our research further showed that GPC-1 acted as an oncogene and promoted cancer progression in various tumors, especially HCC. The previous study showed that the expression of GPC-1 was upregulated in HCC tissues, cells and serum of patients and silencing GPC-1 expression attenuated cell proliferation on HepG2 cell, but more functions and potential mechanisms of GPC-1 still needed to be explored^36,37^. Although the bioinformatic analysis WGCNA analysis also give us some strong insights into GPC-1 in liver cancer, more biological experimentation is needed to verify our findings and promote clinical utility. Therefore, we performed functional experiments in HCC cell lines to further investigate the potential role of GPC-1 through cell proliferation assay, colony formation assay, apoptosis assay, and wound healing assay. The results of the colony formation assay and cell proliferation monitoring assay suggested that GPC-1 significantly affected cell viability and proliferation in 97H and HUH7, and the knockdown of GPC-1 could effectively promote cell apoptosis, while overexpression of GPC-1 promoted proliferation and inhibited apoptosis in liver cancer cell lines. Meanwhile, wound healing assay indicates that GPC-1 expression has no significant effect on cell migration in 97H and HUH7.

We investigated and integrated information from different databases and performed functional validation in HCC cell lines, although there are still many unknowns in the mechanism research in common cancer. Nowadays, Jing Li and his colleagues found that GPC-1 promoted aggressive proliferation by regulating the PTEN/Akt/β-catenin pathway in esophageal squamous cell carcinoma^38^. Other studies also reported that GPCs could interact with Wnt-3a or TGF-β by co-immunoprecipitation^39,40^. These findings may provide fundamental knowledge for mechanistic studies and medical treatments. In our study, WGCNA analysis was also used to elucidate its biological function and interacting molecules, and the results showed that GPC-1 mediated biological functions through multiple pathways such as the P13K-Akt signaling pathway, TNF signaling pathway, and hedgehog signaling pathway, and the expression of the GPC-1 was closely related to GPCR, Akt, P13K, and other molecules. Even though the finding showed that GPC-1 expression was correlated with some related genes in HCC, we were unsure whether GPC-1 interacts with these genes to be involved in the occurrence and development of cancer. Combined with adjacent meta co-expression network in HCCDB database, we identified Akt as a key GPC-1 related molecule and verified that AKT and phosphorylated‐AKT (ser473) expression was increased when GPC-1 was upregulated in 97H and HUH7 cells, and the opposite trend occurred when GPC-1 was down-regulated. Although we did not go into this area in depth, it will provide a direction for future research. All in all, GPC-1 has abstracted more and more attention, and the bioinformatic analysis provide us some meaningful insights of GPC-1 in cancers and some function experiments imply the role of GPC-1 in HCC.

## Conclusion

In conclusion, GPC-1 was highly expressed in HCC, and its expression was significantly negatively correlated with survival and positively correlated with the immune score of immune cells. The expression of GPC-1 was increasing with the clinical stage. Functional experiments suggested that GPC-1 promoted proliferation and inhibited apoptosis in HCC cell lines. WGCNA analysis and HCCDB database revealed that Akt acted as a key molecule related to GPC-1, influencing biological functions and regulating cell malignant behaviors via the AKT signaling pathway. Our findings indicated that GPC-1 was an important oncogene and might be a novel therapeutic target of HCC.

### Supplementary Information

Supplementary Information 1.

Supplementary Information 2.

## Data Availability

The datasets generated and analyzed during the current study are publicly available. This data can be found here: The Cancer Genome Atlas (https://cancergenome.nih.gov), cBioPortal (http://cbioportal.org), TCGA database (https://cancergenome.nih.gov), Genotype-Tissue Expression database (https://commonfund.nih.gov/GTEx/data), Cancer Cell Line Encyclopedia database (https://portals.broadinstitute.org/ccle), California Santa Cruz Xena website (https://xena.ucsc.edu), Tumor Immune Estimation Resource (https://cistrome.shinyapps.io/timer/), UALCAN (http://ualcan.path.uab.edu/index.html), and HCCDB data (https://www.hccdatasph.cn/app/ihga).

## Abbreviations

GPCs: Glypicans
WGCNA: Weighted gene co-expression network analysis
KEGG: Kyoto Encyclopedia of Genes and Genomes
GO: Gene ontology
CC: Cellular component
ACC: Adrenocortical carcinoma
BRCA: Breast invasive carcinoma
CHOL: Cholangiocarcinoma
ESCA: Esophageal carcinoma
HNSC: Head and neck squamous cell carcinoma
KIRC: Kidney renal clear cell carcinoma
LAML: Acute myeloid leukemia
LIHC: Liver hepatocellular carcinoma
LUSC: Lung squamous cell carcinoma
OV: Ovarian serous cystadenocarcinoma
PCPG: Pheochromocytoma and Paraganglioma
READ: Rectum adenocarcinoma
SKCM: Skin cutaneous melanoma
TGCT: Testicular germ cell tumors
THYM: Thymoma
UCS: Uterine carcinosarcoma
HS: Heparan sulfate
TIMER: Tumor immune estimation resource
OS: Overall survival
BP: Biological process
MF: Molecular function
BLCA: Bladder urothelial carcinoma
CESC: Cervical and endocervical cancers
DLBC: Lymphoid neoplasm diffuse large B-cell lymphoma
GBM: Glioblastoma multiforme
KICH: Kidney chromophobe
KIRP: Kidney renal papillary cell carcinoma
LGG: Brain lower grade glioma
LUAD: Lung adenocarcinoma
MESO: Mesothelioma
PAAD: Pancreatic adenocarcinoma
PRAD: Prostate adenocarcinoma
SARC: Sarcoma
STAD: Stomach adenocarcinoma
THCA: Thyroid carcinoma
UCES: Uterine corpus endometrial carcinoma
UVM: Uveal melanoma
