# Supplementary material for: Pan-cancer analysis and the oncogenic role of Glypican 1 in hepatocellular carcinoma
Source: Sci Rep. 2024 Jul 9;14:15870. doi: 10.1038/s41598-024-66838-9 (PMC11233571; doi:10.1038/s41598-024-66838-9)
Supplement: Supplementary file 1 — Supplementary Information 1. [file 41598_2024_66838_MOESM1_ESM.docx]

***Supplementary Material***

**Pan-cancer analysis and the oncogenic role of Glypican 1 in hepatocellular carcinoma**

Li Cao ^2, †^, Fang Li^2, †^, Shuang Cai^2^, Jinyuan Zhang^2^, Chen Guo^2^, Sadiq Ali^2^, Jing Zhou^2^, Xintao Jing^2^, Xiaofei Wang^3, *^, Yannan Qin ^2, *^, Fei Wu^1, 2, *^

^1^ Department of Oncology, The Second Affiliated Hospital of Xi’an Jiaotong University, Xi’an, Shaanxi, 710061, P. R. China

^2^ Department of Cell Biology and Genetics, School of Basic Medical Sciences, Xi’an Jiaotong University, Xi’an, Shaanxi, 710061, P. R. China

^3^ Biomedical Experimental Center of Xi'an Jiaotong University, Xi’an, Shaanxi, 710061, P. R. China

^*^Corresponding authors

Address correspondence to:

Fei Wu, Comprehensive Breast Care Center, The Second Affiliated Hospital of Xi’an Jiaotong University, Xi’an, Shaanxi, 710061, P. R. China. E-mail:[wufei931105@xjtu.edu.cn](mailto:wufei931105@xjtu.edu.cn). ORCID ID: <https://orcid.org/0000-0001-5388-0003>.

**Table S1** Sequences of siRNA

| Name | Sequence | |
| --- | --- | --- |
| negative siRNA (NC-siRNA) sense | 5′-UUCUCCGAACGUGUCACGUTT-3′ |  |
| negative siRNA (NC-siRNA) antisense | 5′- ACGUGACACGUUCGGAGAATT-3′ | |
| GPC1 siRNA-1 sense | 5′- AGCAGAUCAUGCAGCUGAATT-3′ | |
| GPC1 siRNA-1 antisense | 5′- UUCAGCUGCAUGAUCUGCUTT-3′ | |
| GPC1 siRNA-2 sense | 5'- AGCUGAAGAUCAUGACCAATT-3' | |
| GPC1 siRNA-2 antisense | 5'- UUGGUCAUGAUCUUCAGCUTT-3' | |

**Table S2** Primer sequence used for qRT-PCR

| Gene | Sequence |
| --- | --- |
| GPC1-F | 5'- TGAAGCTGGTCTACTGTGCTC-3' |
| GPC1-R | 5'- CCCAGAACTTGTCGGTGATGA-3' |
| GAPDH-F | 5'-GCCGTATCGCTCAGACAC-3' |
| GAPDH-R | 5'-GCCTAATACGACCAAATCC-3' |

**Table S3** Information on antibodies used for the correlation analysis

| Antibody | WB | Specificity | Company | Cat No. |
| --- | --- | --- | --- | --- |
| GPC1 | 1:5000 | Rabbit Monoclonal | Sigma | ZRB1374-4X25UL |
| β-actin | 1:5000 | Rabbit Monoclonal | Proteintech | 81115-1-RR |
| Bax | 1:4000 | Rabbit Monoclonal | Proteintech | 50599-2-Ig |
| Bcl-2 | 1:1000 | Mouse | Cell Signaling Technology | 15071 |
| AKT | 1:1000 | Rabbit Monoclonal | Proteintech Group, | 60203-2-Ig |
| p-AKT(Ser473) | 1:1000 | Rabbit Monoclonal | Cell Signaling Technology | 4060S |
| HRP-conjugated Affinipure Goat Anti-Rabbit IgG(H+L) | 1:10000 |  | Proteintech | SA00001-2 |
| HRP-conjugated Affinipure Goat Anti-Mouse IgG(H+L) | 1:50000 |  | Proteintech | SA00001-1 |

**
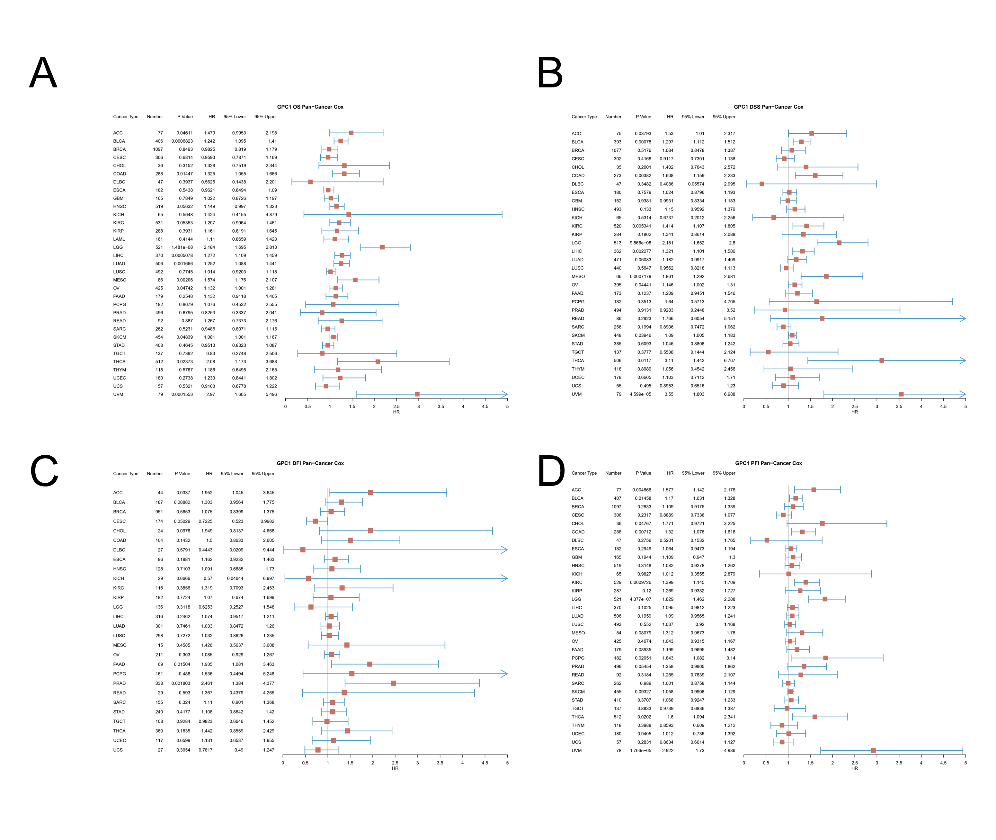
**

**Figure S1** The correlation between GPC-1 expression and COX regression in pan-cancer. (A) OS, (B) DSS, (C) DFI, and (D) PFI. (*P < 0.05, **P < 0.01, ***P < 0.001).


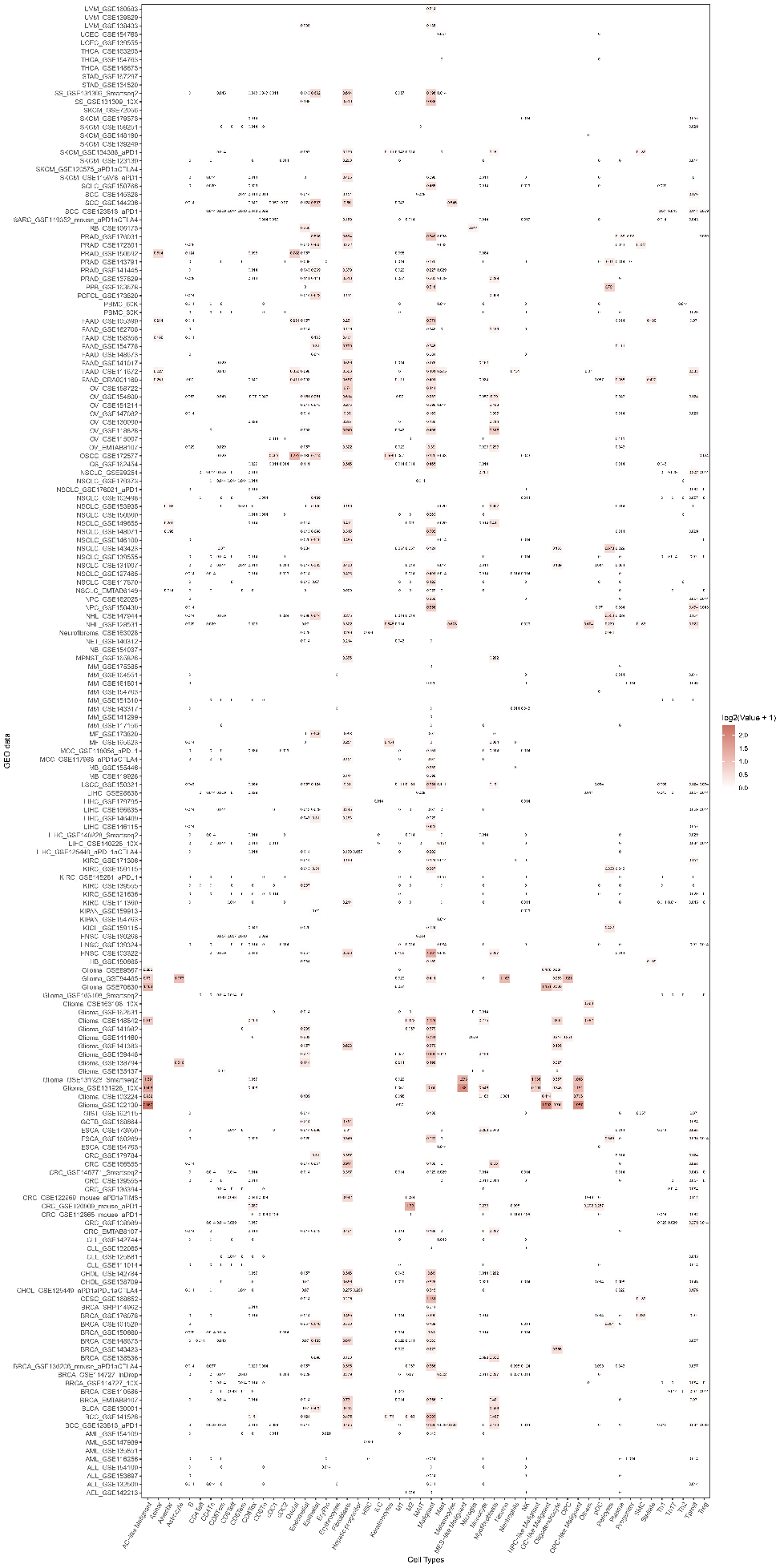


**Figure S2** Summary of GPC-1 expression of 33 cell types in 176 single cell datasets


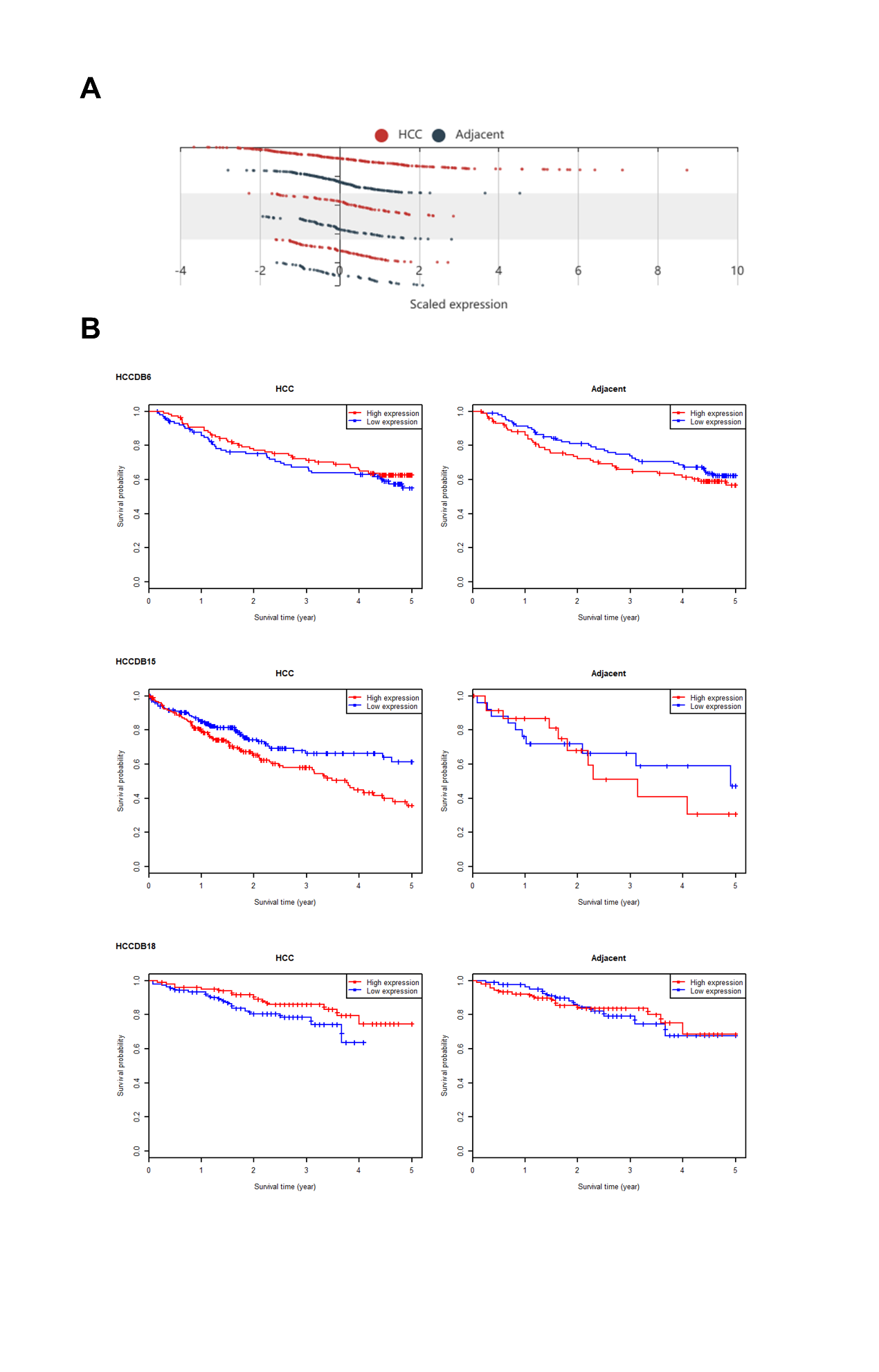


**Figure S3** GPC-1 analysis based on HCCDB database. (A) GPC-1 expression in HCC samples compared with adjacent tissues. (B) The correlation between GPC-1 expression and survival in HCCDB database.


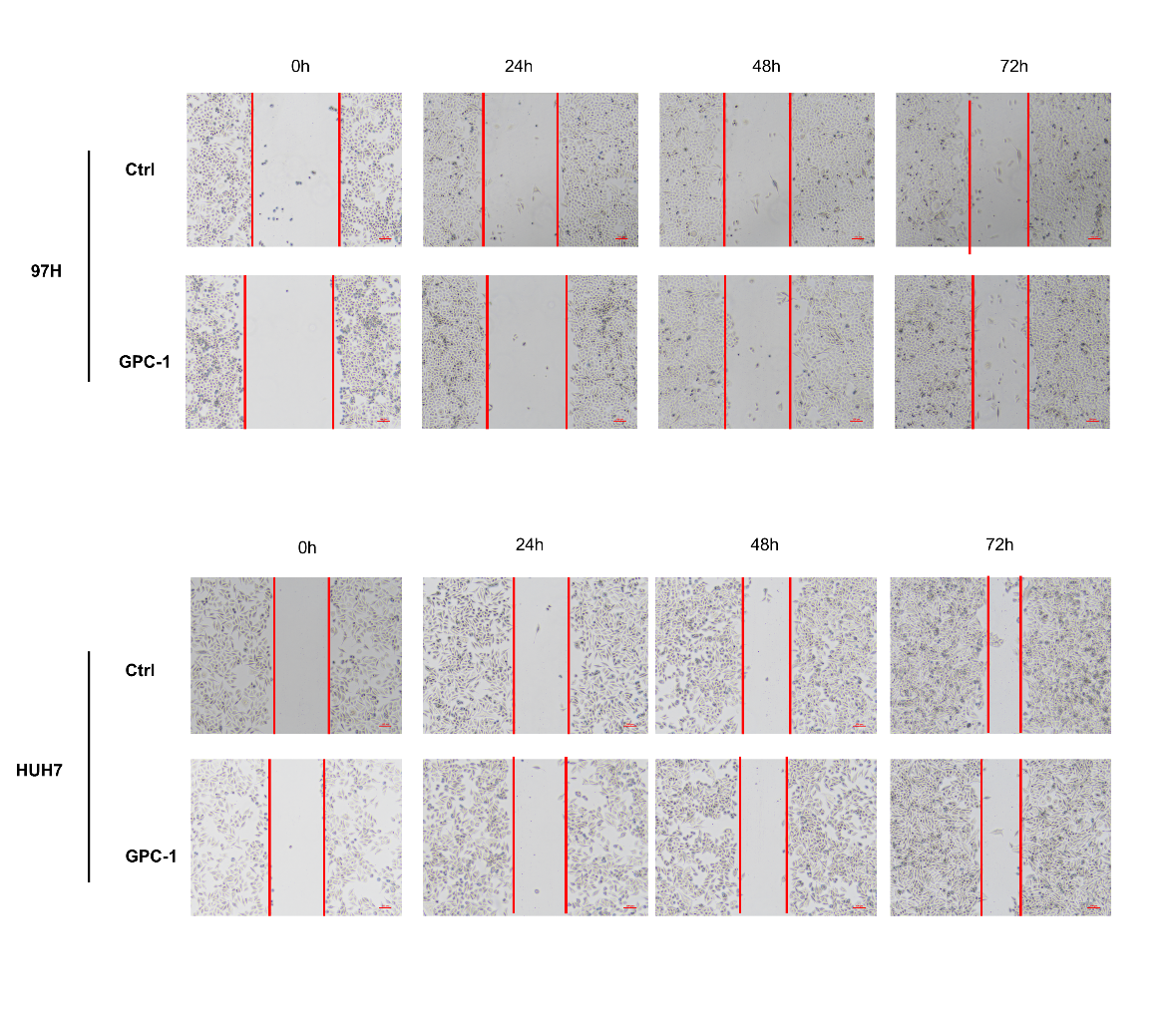
 **Figure S4** 97H (A) and HUH7(B) cells wound assays


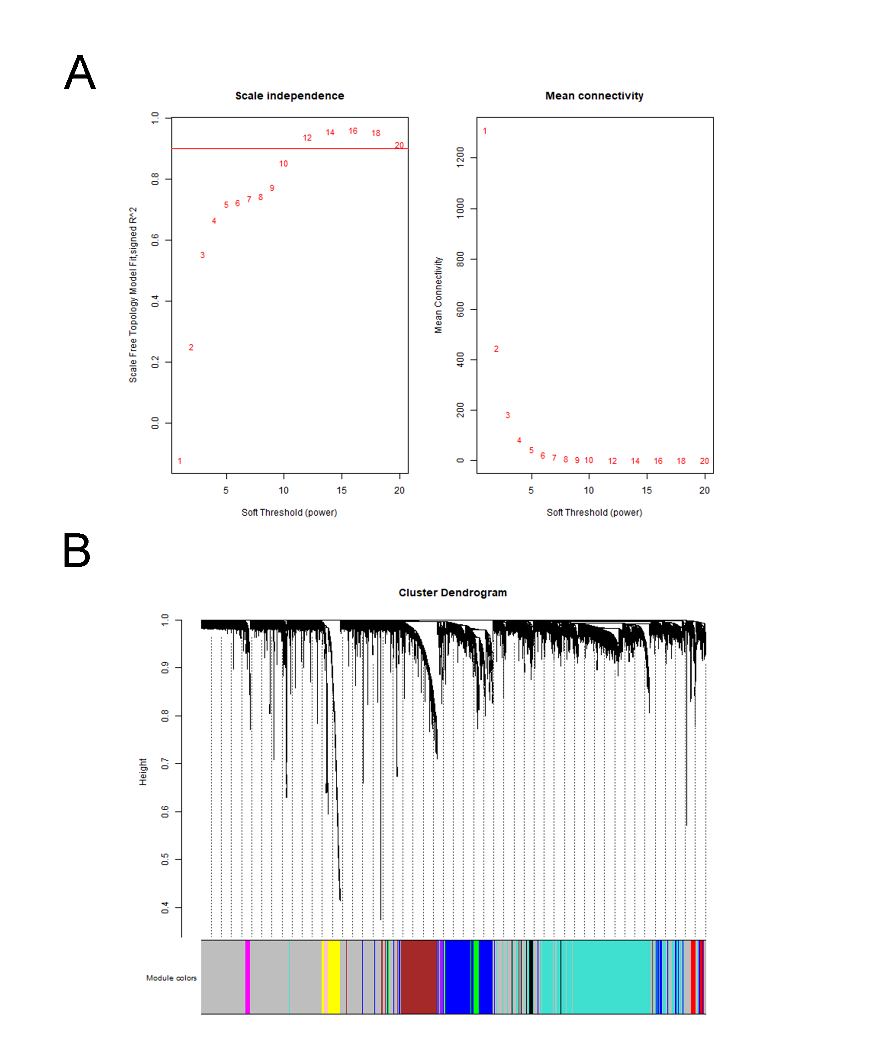


**Figure S5 Functional Enrichment and Pathway Analysis from WGCNA.** (A)Determination of soft-threshold power in the WGCNA. (B) Clustering dendrogram of genes, with dissimilarity based on topological overlap, together with assigned module colors.

**Figure S6** **The uncropped images of western blotting.**

**
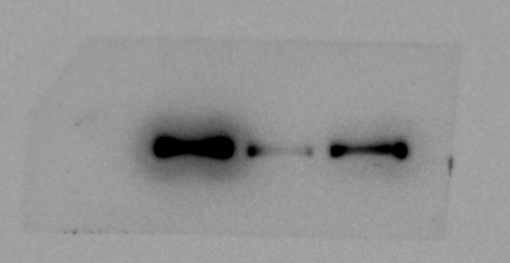

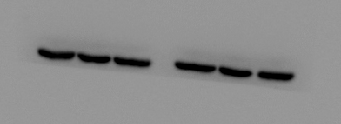
FIGURE 7C**

150KD

70KD

100KD

β-actin

97H

siGPC1-2

siGPC1-1-1

siCtrl

1.
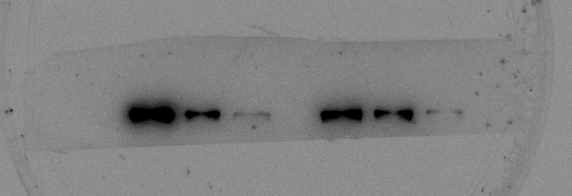

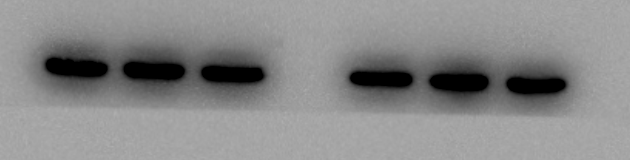


150KD

70KD

100KD

GPC1

40KD

70KD

50KD

β-actin

97H

siGPC1-2

siGPC1-1-1

siCtrl

42KD

GPC1


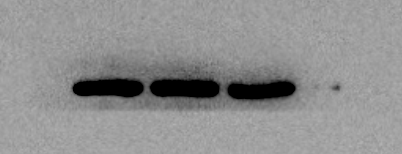

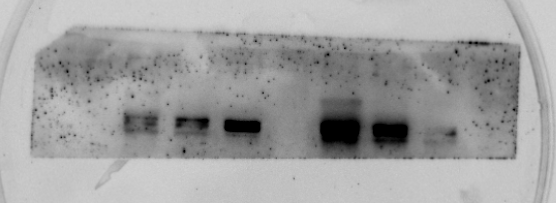

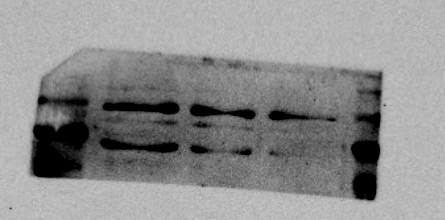

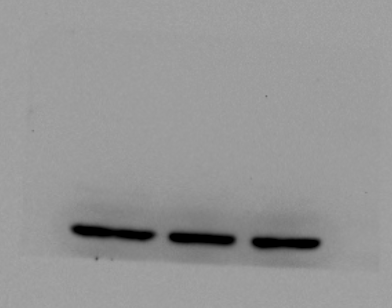


siCtrl

siGPC1-1-1

siGPC1-2

HUH7

β-actin

50KD

40KD

70KD

100KD

150KD

250KD

GPC1

GPC1

150KD

70KD

100KD

β-actin

42KD

97H

siGPC1-2

siGPC1-1-1

siCtrl


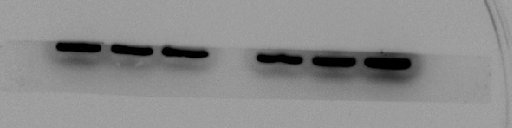

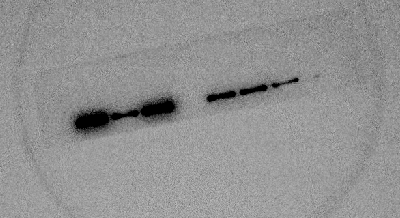


siGPC1-1-1

siGPC1-2

β-actin

42KD

GPC1

86KD

siCtrl


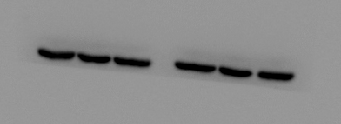

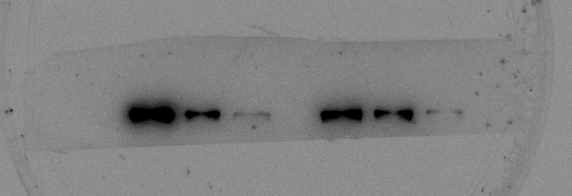


β-actin

siCtrl

siGPC1-1-1

siGPC1-2

GPC1

HUH7

HUH7


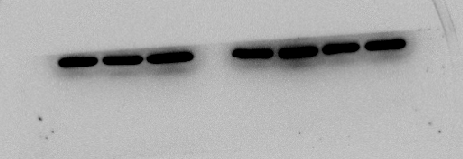

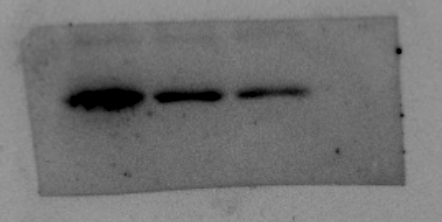

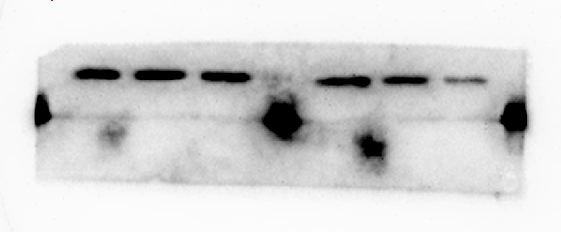

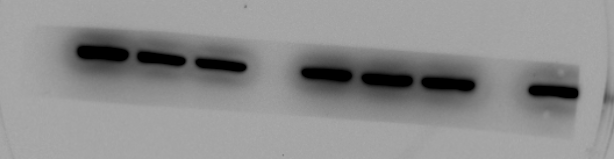

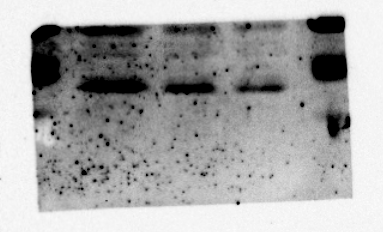

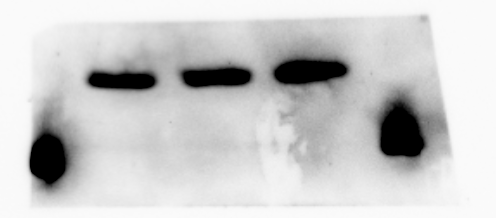
**FIGURE 7F**

42KD

β-actin

Bcl-2

25KD

40KD

35KD

Bax

97H

siGPC1-2

siGPC1-1-1

siCtrl

15KD

25KD

20KD

siCtrl

50KD

40KD

β-actin

Bcl-2

25KD

40KD

35KD

Bax

15KD

25KD

20KD

97H

siGPC1-2

siGPC1-1-1


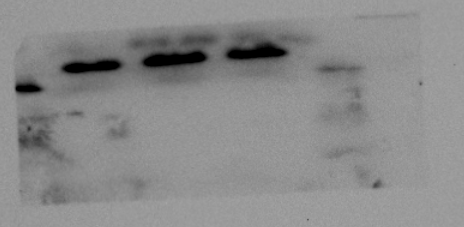

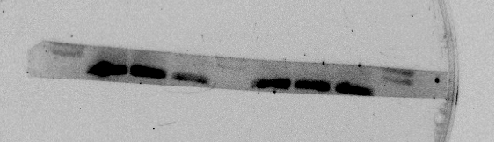

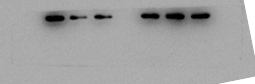

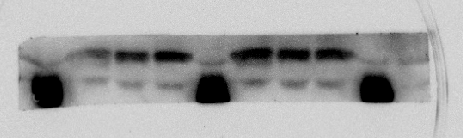

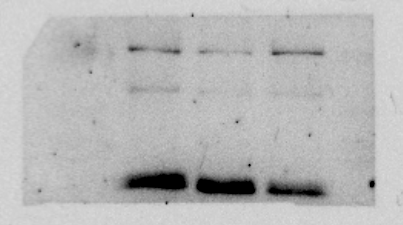

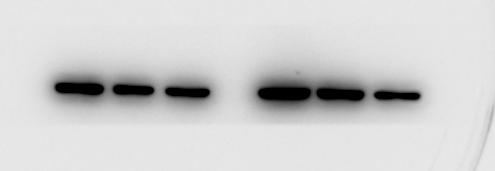


Bax

siCtrl

siGPC1-1-1

siGPC1-2

HUH7

20KD

25KD

15KD

25KD

35KD

Bcl-2

β-actin

50KD

40KD

Bcl-2

Bax

20KD

25KD

siCtrl

siGPC1-1-1

siGPC1-2

97H

35KD

40KD

25KD

50KD

70KD

50KD

40KD

70KD

β-actin


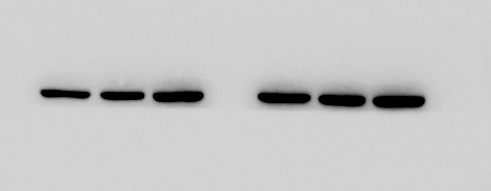

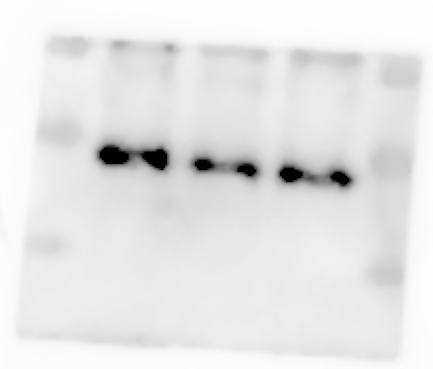

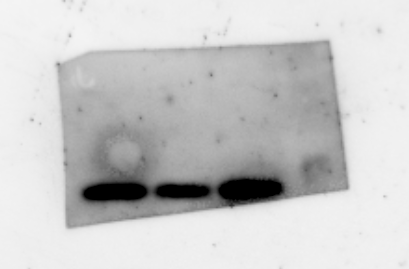

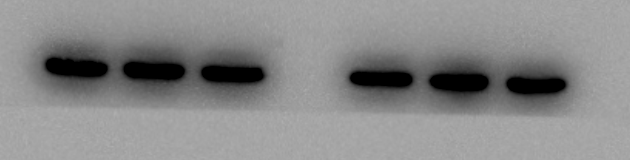

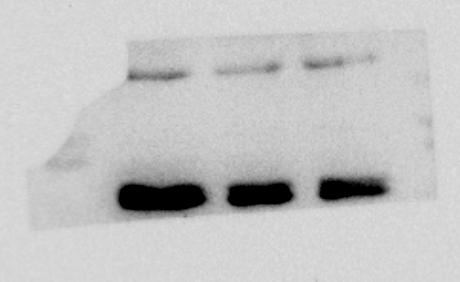

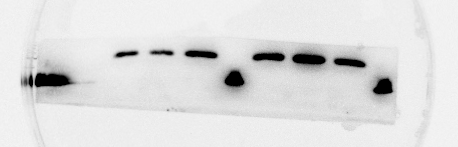


42KD

β-actin

40KD

35KD

25KD

Bcl-2

HUH7

siGPC1-2

siGPC1-1-1

siCtrl

25KD

20KD

Bax

Bax

42KD

β-actin

Bcl-2

25KD

35KD

40KD

50KD

HUH7

siGPC1-2

siGPC1-1-1

siCtrl

15KD

25KD

20KD

**FIGURE 8A**


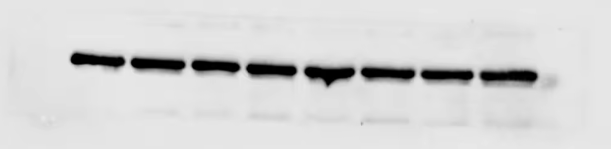


Ctrl

97H

β-actin

40KD

50KD

GPC1


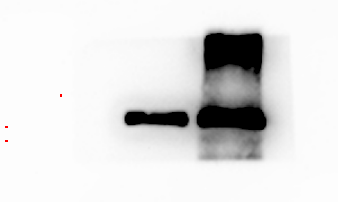

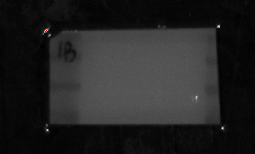


GPC1

70KD


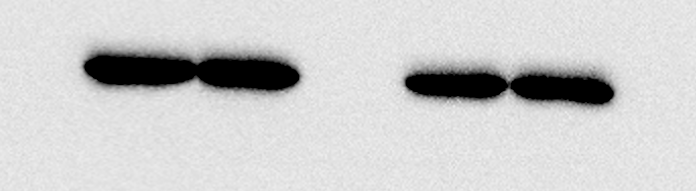


Ctrl

97H

42KD

β-actin

GPC1


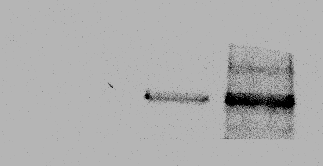

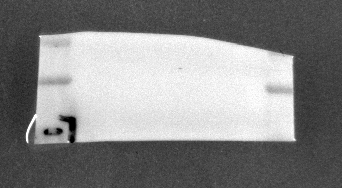


70KD

100KD

150KD

GPC1

150KD


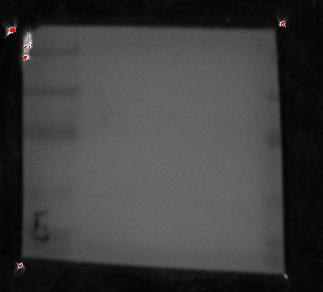

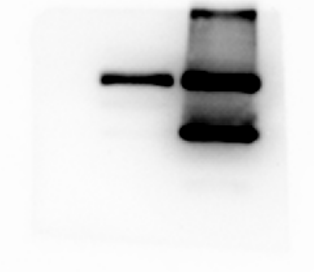

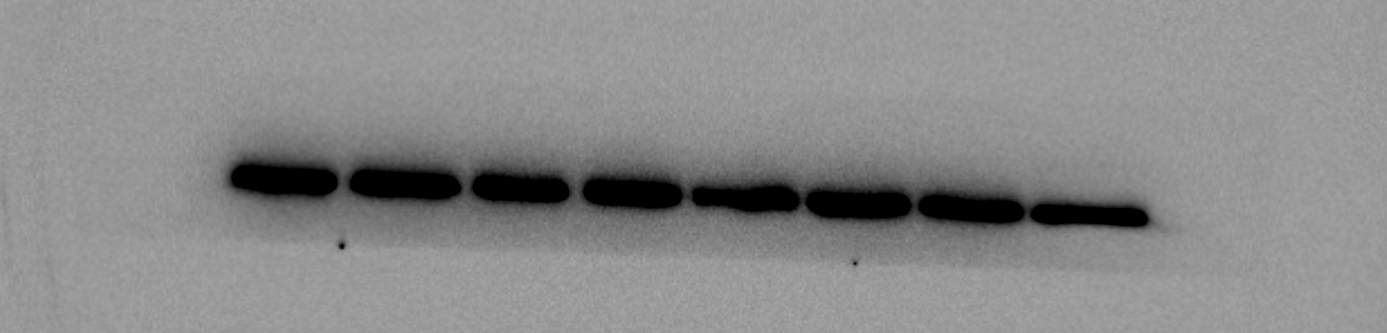

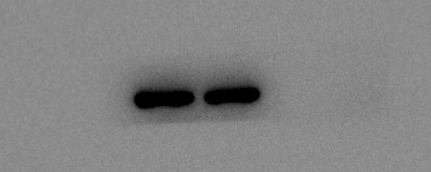

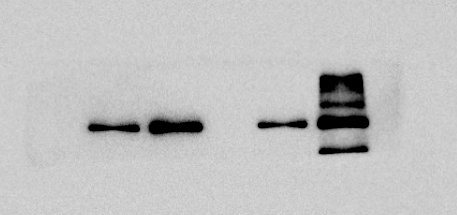

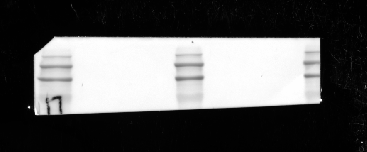

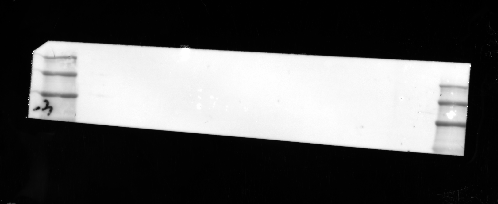

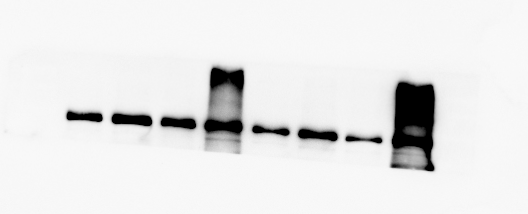

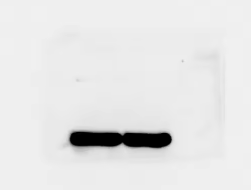


40KD

50KD

70KD

100KD

GPC1

HUH7

Ctrl

50KD

40KD

β-actin

Ctrl

HUH7

GPC1

β-actin

250KD

150KD

100KD

70KD

GPC1

100KD

70KD

150KD

250KD

GPC1

β-actin

100KD

70KD

50KD

40KD

GPC1

97H

Ctrl

100KD

GPC1


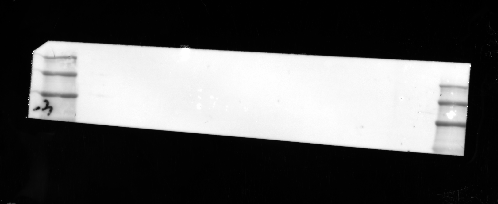

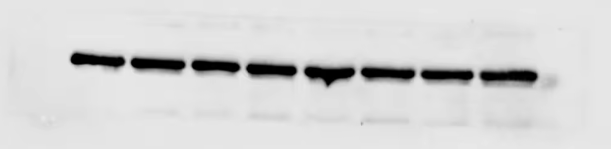

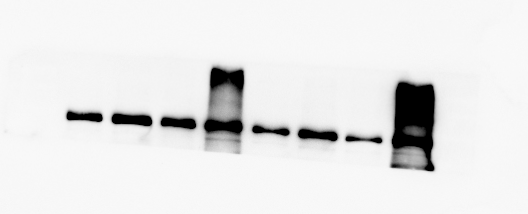


250KD

150KD

70KD

100KD

β-actin

40KD

50KD

Ctrl

HUH7

GPC1

GPC1

**FIGURE 8D
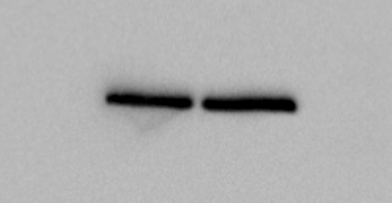

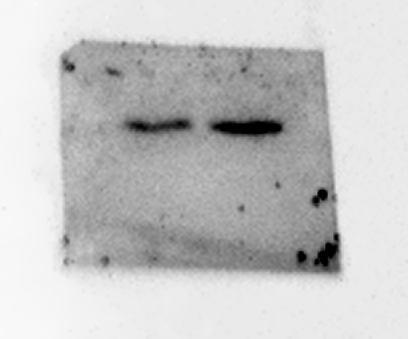

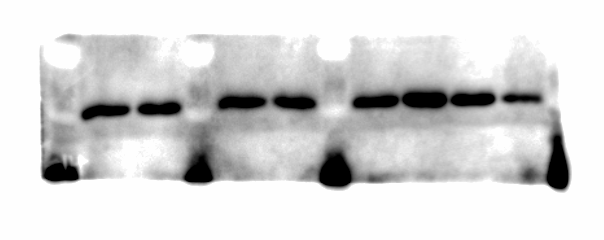

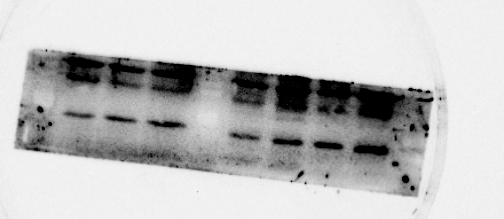

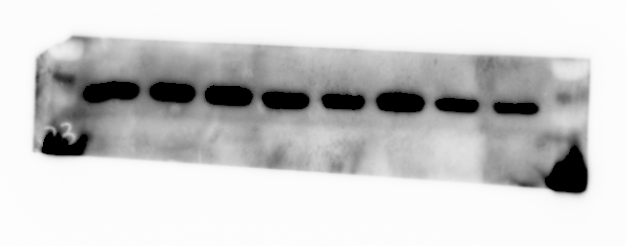

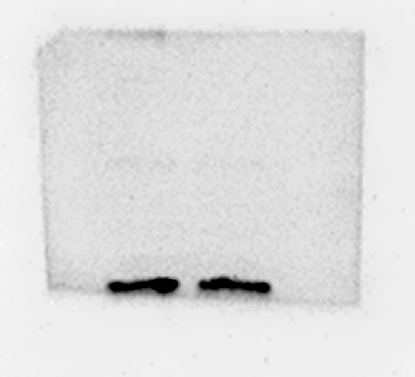

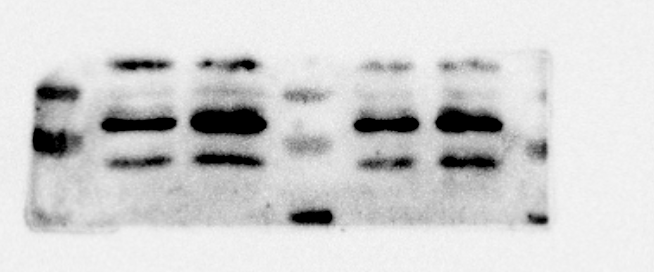

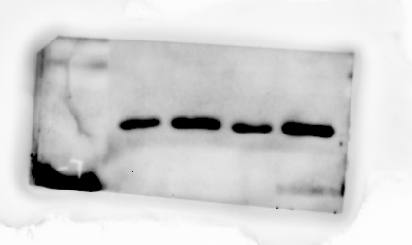

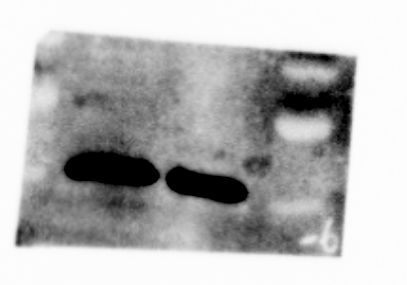

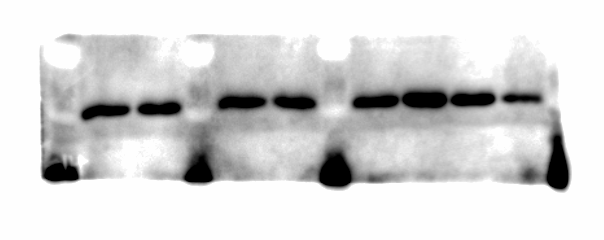

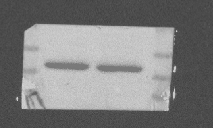

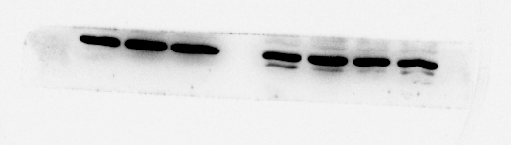
**

42KD

β-actin

Bcl-2

30KD

25KD

20KD

15KD

20KD

25KD

Bax

GPC1

HUH7

Ctrl

Ctrl

97H

GPC1

20KD

25KD

30KD

Bcl-2

Bax

25KD

20KD

15KD

β-actin

42KD

97H

GPC1

Ctrl

97H

GPC1

20KD

25KD

30KD

Bcl-2

20KD

25KD

30KD

Bcl-2

35KD

25KD

20KD

Bax

25KD

20KD

15KD

Bax

β-actin

40KD

50KD

35KD

70KD

β-actin

42KD

Ctrl


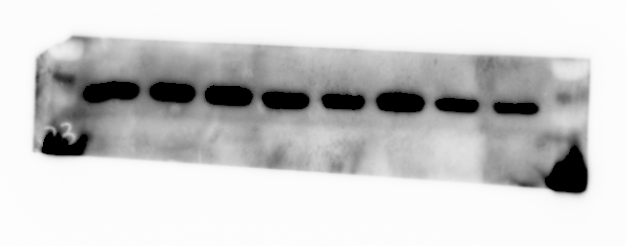

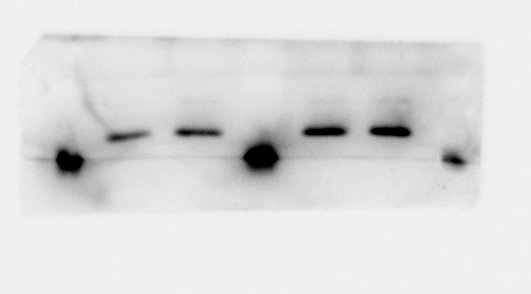

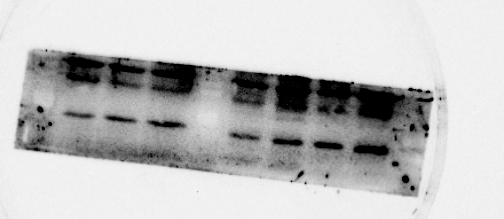

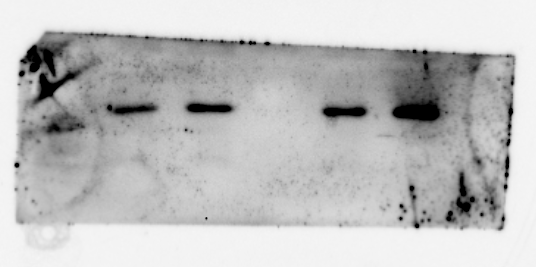

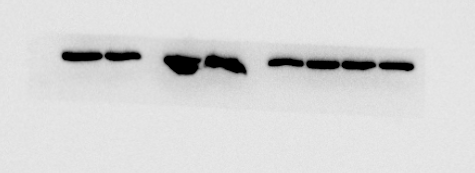

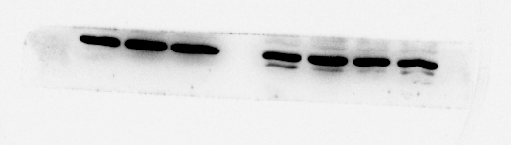


Bax

25KD

20KD

15KD

Ctrl

HUH7

GPC1

Bax

25KD

20KD

Ctrl

HUH7

GPC1

20KD

25KD

30KD

Bcl-2

20KD

25KD

30KD

Bcl-2

β-actin

40KD

50KD

β-actin

42KD


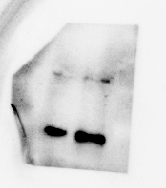

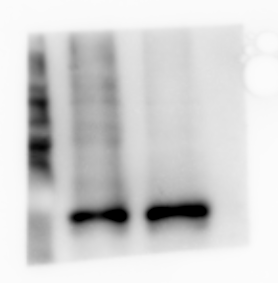

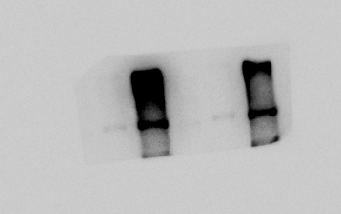

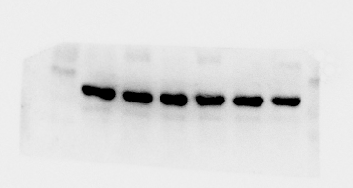
**FIGURE 9G(1)**

50KD

70KD

P-AKT(ser473)

100KD

50KD

70KD

AKT

86KD

GPC1

97H

Ctrl

42KD


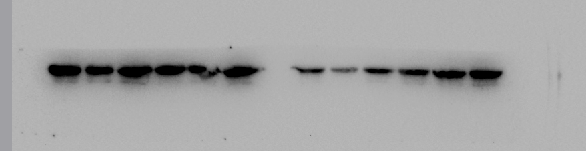

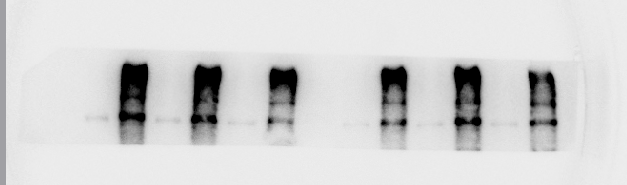

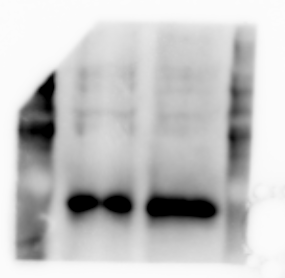

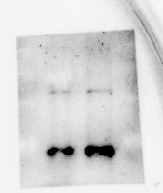


Ctrl

97H

GPC1

42KD

86KD

70KD

50KD

100KD

70KD

50KD

100KD


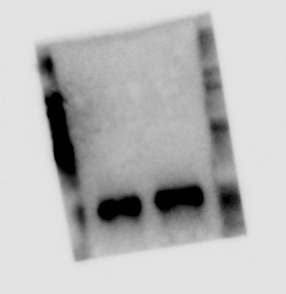

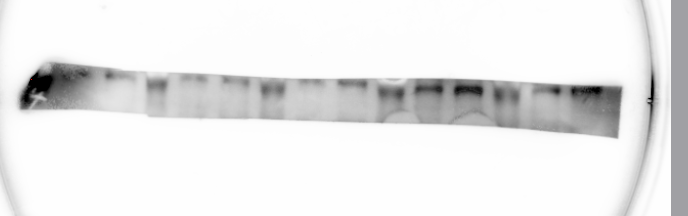

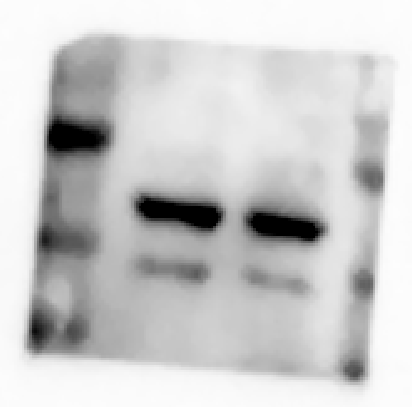

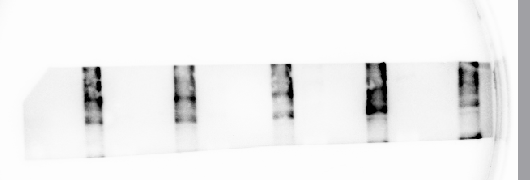


97H

GPC1

Ctrl

AKT

56KD

50KD

70KD

42KD

β-actin

GPC1

86KD

Ctrl

97H

GPC1

P-AKT (ser473)

GPC1

β-actin

P-AKT(ser473)

β-actin

GPC1

AKT


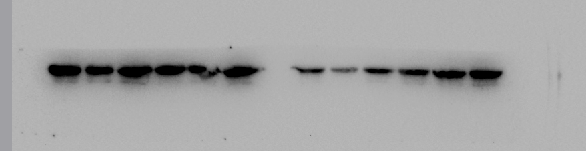

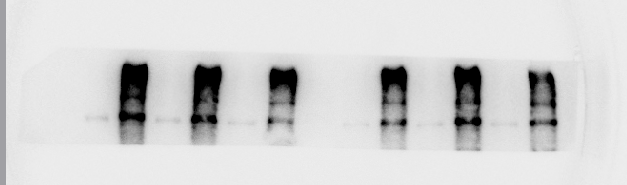

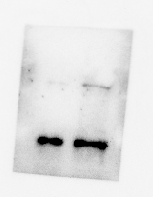

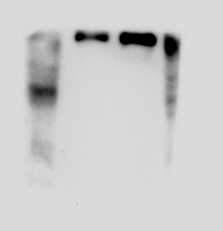

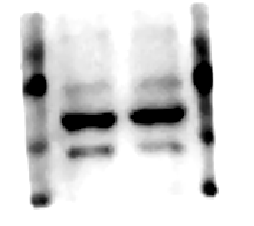

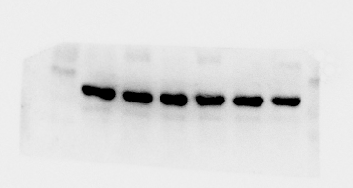

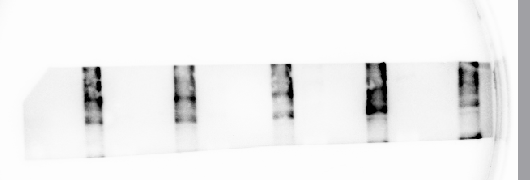

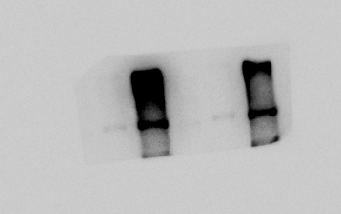

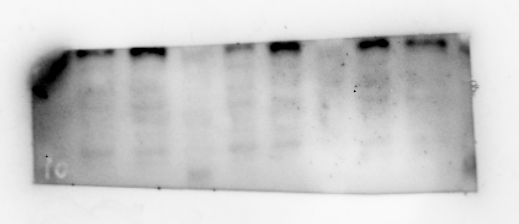

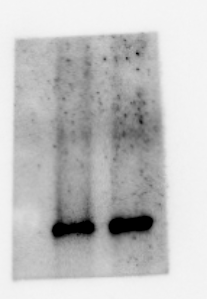

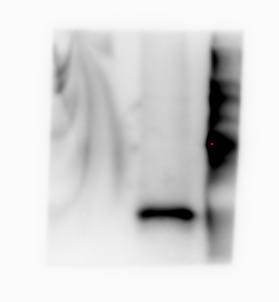

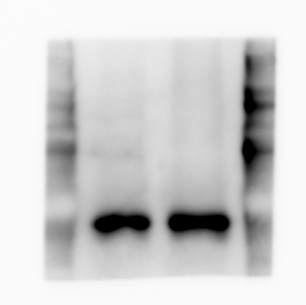


β-actin

GPC1

Ctrl

HUH7

GPC1

Ctrl

42KD

86KD

P-AKT(ser473)

50KD

70KD

50KD

70KD

AKT

GPC1

HUH7

Ctrl

42KD

β-actin

42KD

β-actin

86KD

GPC1

86KD

GPC1

50KD

70KD

P-AKT(ser473)

50KD

70KD

P-AKT(ser473)

50KD

70KD

AKT

50KD

70KD

AKT

GPC1

HUH7

Ctrl

GPC1

HUH7

Ctrl

**FIGURE 9G (2)**


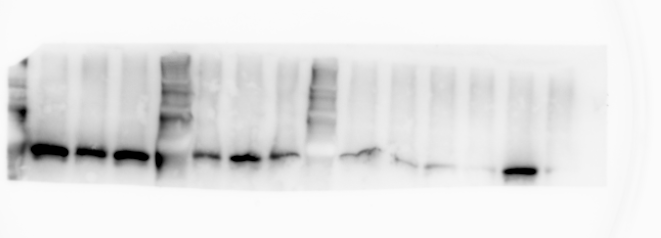

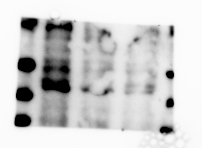

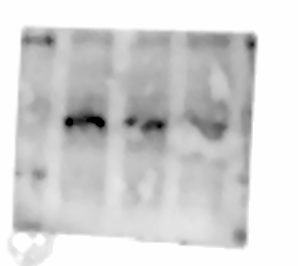

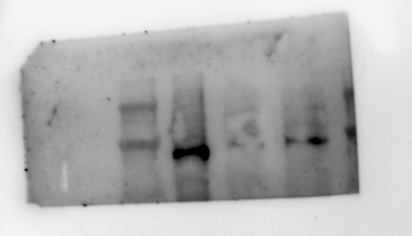

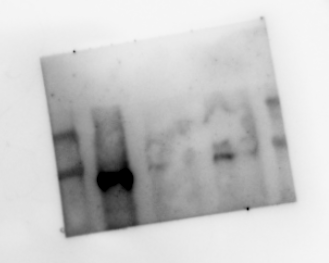


P-AKT(ser473)

P-AKT(ser473)

siCtrl

siGPC1-1-1

siGPC1-2

97H

AKT

70KD

50KD

AKT

70KD

50KD

siCtrl

siGPC1-1-1

siGPC1-2

97H

70KD

50KD

40KD

100KD

70KD

50KD

40KD

100KD

GPC1

86KD

GPC1

86KD

β-actin

42KD

β-actin

42KD

97H

97H

70KD

50KD

56KD

86KD

42KD

siCtrl

siGPC1-1-1

siGPC1-2

97H

AKT

P-AKT(ser473)

GPC1

β-actin

siCtrl

siGPC1-1-1

siGPC1-2

siCtrl

siGPC1-1-1

siGPC1-2

HUH7

AKT

siCtrl

siGPC1-1-1

siGPC1-2

HUH7

P-AKT(ser473)

70KD

50KD

40KD

100KD

70KD

50KD

40KD

100KD

GPC1

86KD

150KD

β-actin

42KD

40KD

50KD

70KD

35KD

100KD

70KD

70KD

50KD

100KD

AKT

P-AKT(ser473)

GPC1

β-actin

HUH7

HUH7

56KD

siCtrl

siGPC1-1-1

siGPC1-2

70KD

86KD

42KD

AKT

P-AKT(ser473)

GPC1

β-actin

siCtrl

siGPC1-1-1

siGPC1-2
